# Current challenges and future directions in the green synthesis of nanoparticles using fungi

**DOI:** 10.1007/s00449-026-03430-7

**Published:** 2026-09-23

**Authors:** Angélica Ribeiro Soares, Giovani Pavoski, Denise Crocce Romano Espinosa, WenBing Yin, Marcela dos Passos Galluzzi Baltazar

**Affiliations:** 1 Chemical Engineering Department, University of São Paulo, Rua do Lago, 250 – 2º andar, São Paulo, 05508-080 Brazil; 2 State Key Laboratory of Microbial Diversity and Innovative Utilization, Institute of Microbiology, Chinese Academy of Sciences, Beijing, 100101 PR China

**Keywords:** Green nanotechnology, Mycogenic nanoparticles, Metal nanoparticles, Multi-omics, Bioprocess

## Abstract

Fungi have historically played a significant role in advancing biotechnology and are now increasingly recognized as flexible platforms for eco-friendly nanoparticle production. Nonetheless, progress has been hindered by inconsistent methodologies, limited mechanistic understanding, and reproducibility issues. Unlike earlier reviews, this study comprehensively analyzes a decade of research (2015–2025) to link fungal species diversity, biosynthesis pathways, and experimental setups to nanoparticle formation. It highlights the crucial roles of enzymatic activity and secreted metabolites in determining nanoparticle shape and stability. The study demonstrates that variables such as culture medium, pH, incubation period, and downstream processes are not minor technical details but key factors influencing nanoparticle size, morphology, and stability. Additionally, there is a strong taxonomic bias toward a narrow set of filamentous fungi and a material bias toward silver, gold, and zinc, despite the broader biosynthetic potential of the fungal kingdom and the growing relevance of unexplored metals, including critical metals (e.g., Nb, Zr, and V). By combining mechanistic insights with advanced transcriptomic, proteomic, metabolomic, and genomic data, myconanotechnology can move toward a predictive framework based on metal stress responses, secreted biomolecules, and redox metabolism. Achieving this shift is critical to improving reproducibility, scaling up production, and developing rational approaches to the creation of fungal nanomaterials for biomedical, environmental, and industrial applications.

**Supplementary Information:**

The online version contains supplementary material available at https://doi.org/10.1007/s00449-026-03430-7.

## Introduction

Nanotechnology is reshaping modern science and technology, driving breakthroughs across medicine, agriculture, engineering, chemistry, and environmental sciences [1–3]. By enabling the manipulation of material at the nanoscale, this technology enhances mechanical strength and improves chemical resistance. For instance, incorporating nanomaterials such as nano-silica and nano-titanium dioxide (TiO₂) into cement matrices improved durability [4]. At the same time, nanomaterials are increasingly applied in hydrometallurgy to optimize metal separation and recovery [5].

**Fig. 1:**
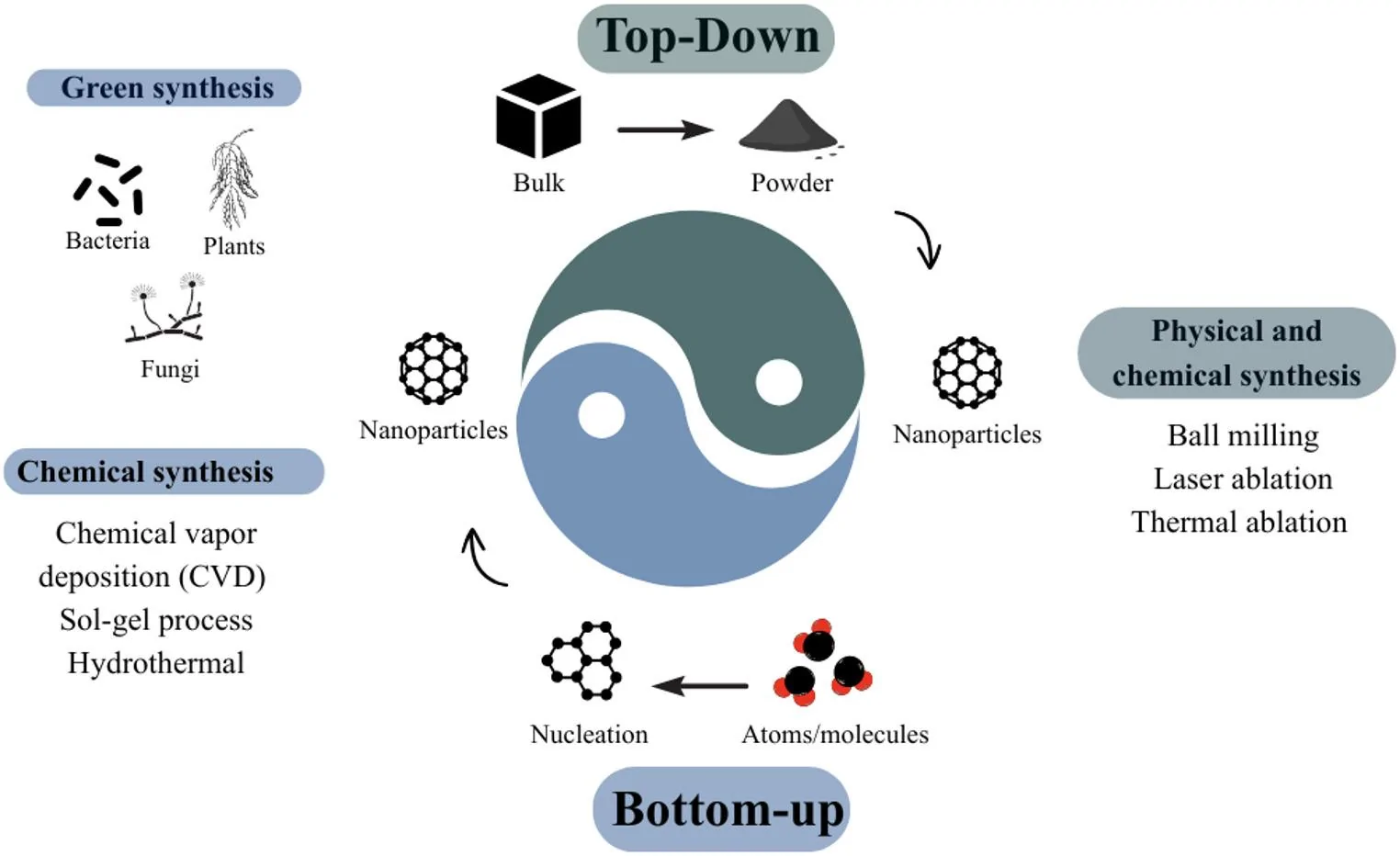
An overview of nanomaterial synthesis approaches. The top-down approach involves forming nanoparticles from bulk materials, whereas the bottom-up approach constructs nanoparticles from molecular precursors

Nanoparticles are typically synthesized via top-down or bottom-up methods (Fig. 1). The top-down method involves breaking down bulk materials into nanoscale particles through ball milling, spraying, thermal, or laser ablation, often combined with stabilizers to prevent aggregation [6]. However, this approach is frequently associated with limitations such as high energy consumption, low surface control, and potential structural defects in the resulting nanoparticles. Conversely, the bottom-up approach assembles atoms or molecules into nanoparticles via chemical processes, such as chemical vapor deposition (CVD), sol-gel processes, and hydrothermal [7]. This method generally enables superior control over particle size, morphology, and surface properties, making it more suitable for advanced nanotechnological applications. Green synthesis has gained prominence in bottom-up methods by avoiding toxic reagents, such as sodium citrate, and leveraging biological systems, including fungi, bacteria, yeasts, and plants, as reducing and stabilizing agents [8–10]. In this context, biomolecules such as proteins, enzymes, polysaccharides, and secondary metabolites play a crucial role in metal ion reduction and nanoparticle capping, enhancing stability and functionality. This eco-friendly approach aligns with global initiatives to mitigate environmental impacts and foster sustainable practices, thereby contributing to the achievement of United Nations Sustainable Development Goals (SDGs) 9 and 12 [11]. Moreover, it reduces hazardous waste generation and improves biocompatibility, which is particularly advantageous for biomedical and environmental applications.

Among biological systems, fungi are particularly promising due to their metabolic versatility and ability to produce enzymes and secondary metabolites that mediate nanoparticle nucleation and stabilization [12–14]. Fungal-mediated synthesis has been successfully demonstrated for various metals, particularly silver, zinc, gold, and copper [15–19]. However, variability in experimental protocols and limited understanding of the underlying molecular mechanisms hinder reproducibility and industrial scalability.

Emerging omics technologies, including transcriptomics, proteomics, and metabolomics, offer transformative potential to address these gaps. By elucidating gene expression dynamics, regulatory networks, and metabolic pathways involved in nanoparticle biosynthesis, these approaches can guide strain selection, culture optimization, and potential genetic or metabolic engineering to enhance yield, specificity, and reproducibility. Analogous strategies have previously advanced biotechnology, such as in the large-scale penicillin production [14], demonstrating the value of mechanistic insight for process optimization.

Recent reviews have addressed various aspects of fungal nanotechnology, including fungal-mediated synthesis routes, physicochemical factors influencing nanomaterial properties, toxicity, scale-up challenges, agricultural applications, and ecological risks [20, 21]. These studies have emphasized challenges related to process optimization, environmental safety, field validation, and regulatory development. However, a systematic assessment that integrates the development of fungal-mediated nanoparticle synthesis with taxonomic and geographic trends, nanoparticle characteristics, experimental practices, and proposed biosynthetic mechanisms over the past decade remains limited. Therefore, this review analyzes studies published between 2015 and 2025 to identify recurring methodological gaps, underexplored research areas, and emerging trends, providing a comprehensive perspective on the current state and future directions of fungal-mediated nanoparticle synthesis.

## Methods

### Search processing

This systematic review followed the Preferred Reporting Items for Systematic Reviews and Meta-Analyses (PRISMA) guidelines [22]. It used the PubMed, Scopus, and Web of Science databases to conduct a comprehensive search for scholarly articles on fungal-derived nanoparticles. The review included publications documented from 2015 to 2025. The Boolean operators were employed to combine relevant keywords, as detailed in Table S1. The search was limited to articles published in English.

### Inclusion/exclusion criteria

Articles were incorporated into the study contingent upon their compliance with the specified criteria: (1) investigating the fungal-mediated nanoparticle synthesis with mechanistic insights; (2) reporting on biological pathways, enzymes, metabolites, or fungal species involved in nanoparticle formation; (3) primary research articles (experimental studies).

Articles were excluded if they exhibited these features: (1) studies lacking mechanistic insights or not focused on fungi; (2) non-peer-reviewed literature (e.g., preprints, thesis dissertations, patents, blogs, or opinion pieces); (3) purely theoretical or computational studies lacking experimental validation and studies focusing on non-fungal organisms (e.g., bacteria, algae, plants) unless fungi are involved; (4) research on nanoparticles synthesized by chemical or physical methods (without fungal mediation); (5) articles not published in English; (6) review articles.

### Quality assessment

Given the absence of validated quality-assessment tools for in vitro fungal biosynthesis studies, a custom checklist was developed based on nanomaterial reporting standards. This checklist evaluated: (i) completeness of experimental parameter reporting (medium composition, pH, incubation time, fungal strain identification); (ii) nanoparticle characterization rigor (size-distribution methodology, morphological analysis, stability assessment); (iii) statistical reporting (number of replicates, variance measures); and (iv) mechanistic evidence quality (controls, reproducibility across studies). Studies were rated as adequate, moderate, or inadequate based on these criteria.

## Results

### Study selection, characteristics and geographic distribution

862 research articles were identified using electronic databases (PubMed = 327, Scopus = 494, Web of Science = 41). After removing 102 duplicates, the titles and abstracts of 760 articles were screened. 609 records were excluded for not meeting the inclusion criteria. Of the 134 assessed for eligibility, 34 were excluded (12 reviews; 22 articles with inadequate quality). Consequently, 100 articles were included in the qualitative synthesis (Table S2). The PRISMA flow diagram (Figure S2) illustrates the selection process. Most studies were published from 2019 onward, indicating growing interest in fungal-mediated nanoparticle biosynthesis. Analysis of the 100 studies included in this review reveals that research efforts are predominantly concentrated in Asia and Latin America. The most frequently represented countries are India (37%), Egypt (19%), China and Saudi Arabia (9%), and Iran and Brazil (each 4%). This distribution underscores the leadership of institutions in advancing green nanotechnologies and fungal biotechnology [23–25].

## Discussions

### Overview of experimental workflow and key reporting parameters

Fungal-mediated nanoparticle synthesis involves a sequence of interconnected biological and physicochemical steps that can be organized into fungal cultivation, preparation of biological material, and exposure to the metal precursor, nanoparticle formation, downstream recovery, and physicochemical characterization. Fungal biomolecules, including enzymes, proteins, polysaccharides, and secondary metabolites, may participate in metal transformation, nucleation, and nanoparticle stabilization [12, 13]. After cultivation, synthesis can proceed through intracellular and extracellular routes. In intracellular synthesis, metal ions interact directly with the fungal biomass, and nanoparticle formation occurs at the cell wall [26]. In extracellular synthesis, secreted biomolecules are recovered in culture filtrates or aqueous extracts and subsequently interact with the metal precursor outside the fungal cells [27].

Experimental conditions in each stage can influence nanoparticle formation and their physicochemical properties. Across the reviewed studies, significant variability was observed in parameters such as temperature, pH, incubation time, and agitation conditions, hampering direct comparison among different protocols. Incomplete reporting further compounds this variability; for instance, pH was reported in only 21% of the studies, with none clearly specifying the initial pH. Additionally, downstream procedures, such as centrifugation conditions, were frequently insufficiently described, particularly concerning temperature, loading volume, and relative centrifugal force. These methodological inconsistencies complicate the discrimination between biological effects and process-related variability, presenting a substantial barrier to standardization and reproducibility in fungal-mediated nanoparticle synthesis.

To address these limitations, a minimum reporting framework for fungal-mediated nanoparticle synthesis is proposed (Table 1). The framework organizes the essential information required for experimental transparency into fungal strain identification, cultivation and biomass preparation, synthesis conditions, downstream processing, nanoparticle characterization, and experimental design. The framework integrates the principal reporting gaps identified in this review with additional information necessary for protocol replication and meaningful interstudy comparison. First adoption could provide a practical basis for more consistent reporting and facilitate the development of reproducible and comparable experimental protocols.

**Table 1:** Minimum reporting framework for fungal-mediated nanoparticle synthesis studies

Category	Minimum information to be reported
Fungal strain identification	Fungal species and strain accession number when available; identification method; geographical or environmental origin of the isolate, when relevant.
Strain maintenance and inoculum preparation	Maintenance medium and conditions; storage conditions; inoculum type (spore suspension or mycelial fragments); method used for inoculum preparation.
Fungal cultivation	Complete culture-medium composition; working volume; cultivation time; temperature; initial and final pH; agitation conditions; growth phase or harvesting point, when determined.
Biomass preparation	Amount or concentration of fungal biomass, specifying wet or dry weight; biomass recovery method; washing solution; washing volume; number and duration of washing cycles.
Cell-free filtrate or fungal extract preparation	Biomass-to-liquid ratio; type and volume of extraction/incubation solution; incubation time; temperature; agitation; filtration or centrifugation conditions.
Synthesis route	Intracellular or extracellular synthesis; type of biological material used (biomass, culture filtrate, aqueous or aqueous filtrate).
Metal precursor	Precursor concentration; preparation/dissolution procedure; precursor volume.
Nanoparticle synthesis conditions	Volume and concentration/amount of fungal biomass or filtrate, total reaction volume; fungal material-to-precursor ratio; reaction time; reaction temperature; initial reaction pH and any pH adjustment; agitation conditions; light exposure, when relevant.
Nanoparticle recovery and purification	Recovery method (e.g. centrifugation, filtration); centrifugation condition reported as relative centrifugal force, time, and temperature; filtration characteristics, washing solution, volume, and number of washing cycles.
Drying and storage	Drying temperature and duration; lyophilization conditions; storage temperature and duration.
Nanoparticle yield and synthesis efficiency	When quantitatively assessed, the method used to determine nanoparticle yield, or metal conversion, should be clearly reported. Recovered nanoparticle mass or concentration may be provided as a measure of product yield, whereas metal-conversion efficiency should be supported by quantitative elemental analysis (e.g. ICP-OES, ICP-MS, or AAS*), including initial and residual metal concentrations when applicable.
Physicochemical characterization	Nanoparticle formation or optical properties; Particle size and size distribution; crystalline phase; elemental composition; surface chemistry; hydrodynamic size and polydispersity, when applicable; zeta potential and colloidal stability, when applicable; Ultraviolet-visible spectroscopy (UV-Vis)
Experimental controls	Appropriate positive or negative controls according to the synthesis route and intended application.
Replication and statistical analysis	Number of independent biological replicates and technical replicates; statistical methods; measures of variability (e.g., standard deviation or standard error)
Functional or application-specific evaluation	Nanoparticle concentration or dose; assay conditions; exposure time; appropriate controls; relationship between physicochemical characteristics and biological, catalytic, environmental, or other application-specific performance.

*ICP-OES, inductively coupled plasma optical emission spectroscopy; ICP-MS, inductively coupled plasma mass spectroscopy; AAS, atomic absorption spectroscopy

### Predominant fungal species

While the fungal kingdom is estimated to comprise over 1.5 million species, only approximately 120,000 have been formally described [28]. This significant knowledge highlights the vast untapped reservoir of fungal biodiversity with potential in green nanoparticle production [29]. Their metabolic versatility and ability to thrive under diverse environmental conditions make fungi especially attractive for sustainable industrial processes. Across the 100 studies reviewed, the genera *Aspergillus* (36%), *Penicillium* (19%), and *Fusarium* (14%) predominated. These filamentous Ascomycota share two critical traits relevant to myconanotechnology: production of diverse bioactive secondary metabolites and ecophysiological resilience supporting scalability [30, 31]. Additionally, their well-established use in industrial fermentation processes facilitates their integration into existing biotechnological platforms, reducing barriers for large-scale nanoparticle production. A species-level overview of the fungal strains employed for the biosynthesis of the predominant nanoparticle materials, together with precursor, morphology, particle size, and corresponding references, is provided in Table S3.

The dominance of these three genera may reflect genuine advantages, but it also indicates research bias toward well-characterized, easily cultivatable taxa. Underrepresented genera may harbor untapped biosynthetic potential, but remain unexplored due to cultivation difficulties or lack of prior characterization. *Aspergillus* species, for instance *A.terreus*,* A. fumigatus*, and *A.niger*, are not only widely distributed and easy to cultivate, but also generate a wide arsenal of compounds, such as butenolides, alkaloids, terpenoids, cytocalasins, and anthraquinone derivatives [32, 33]. These metabolites are directly associated with redox activity and metal-binding capacity, which are essential for nanoparticle biosynthesis. Mechanistically, their extracellular enzymes (e.g., reductases, oxidases) mediate electron transfer to reduce metal ions (e.g., Ag+, Au3+) to their zero-valent states [30], while secreted proteins and polysaccharides simultaneously stabilize the resulting nanoparticles. This metabolic plasticity allows fine-tuning of nanoparticle size and morphology, parameters that directly determine catalytic and biomedical performance.

*Penicillium* species are also well researched, playing a pivotal role as decomposers [32, 33] and in mycotoxin production [34]. It also contributes to biotechnology; species such as *P.roqueforti* and *P. camemberti* are used to food fermentation [35] and in the fermentation processes of cured sausages, as *Penicillium nalvogiense* [36]. Their ability to secrete enzymes (e.g., cellulases, proteases) [37, 38] and bioactive metabolites with anti-inflammatory [39], antimicrobial [40], and cytotoxic [41] activities also translates into effective nanoparticle biosynthesis. Enzymatic hydrolysis can release additional reducing sugars and phenolics from biomass substrates, contributing to the mass of the reaction and enhancing nanoparticle yield and stability.

*Fusarium*, despite being a phytopathogen, produces not only mycotoxins, such as trichothecenes, fumonisins, and zearalenone, but also industrially valuable pigments (e.g., carotenoids) and even pharmacologically active compounds such as vincristine [42–44]. Their amylases, xylanases, and β-glucosidases [45–47] can hydrolyze complex substrates, thereby liberating electron donors that participate in metal-ion reduction. This dual role, biomass valorization and nanoparticle synthesis, offers promising avenues for integrated bioprocess design, improving efficiency and resource utilization.

### Types of nanoparticles biosynthesized by fungi

**Fig. 2:**
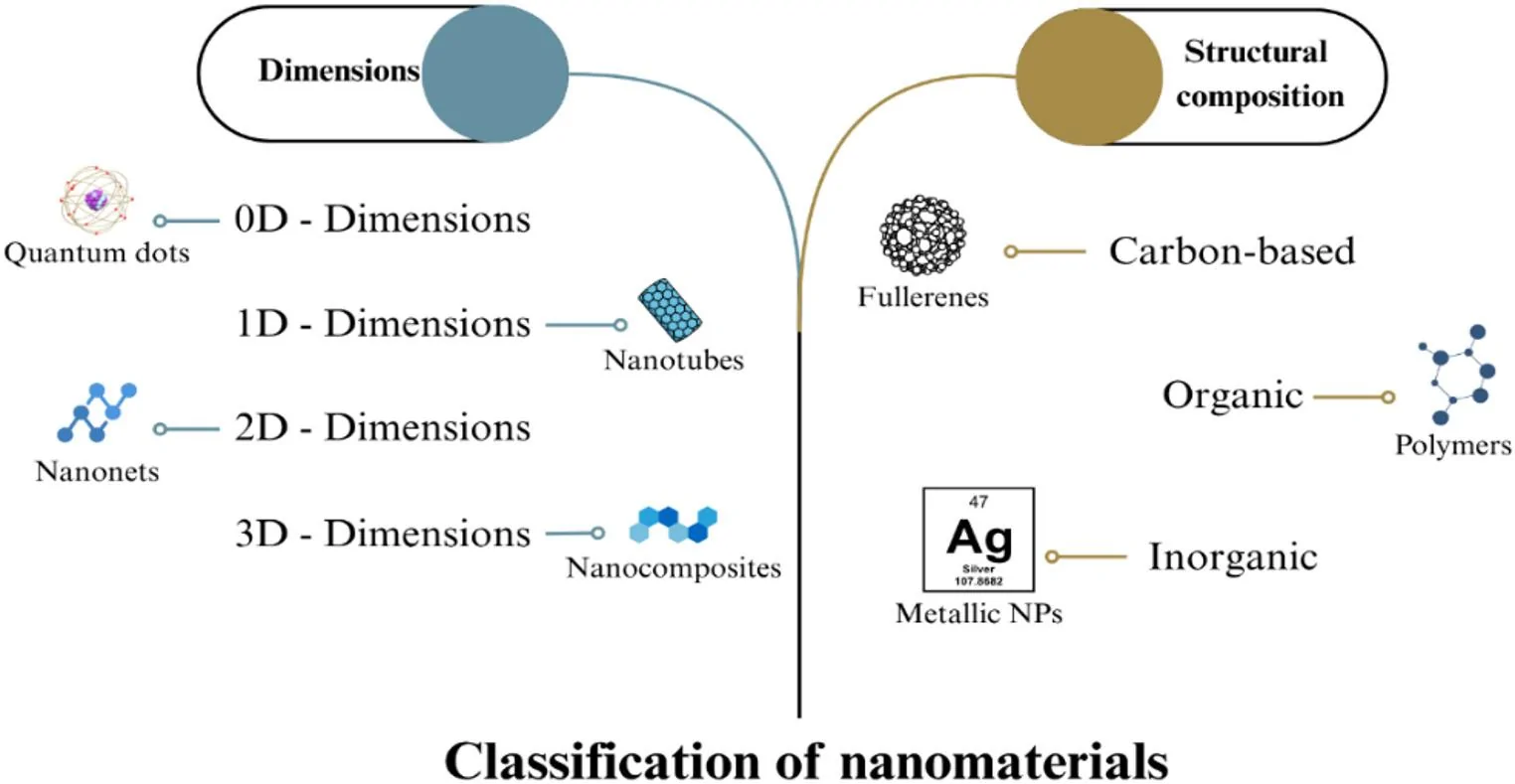
Overview of nanomaterials classification by dimension and composition

Nanomaterials, defined by at least one dimension in the nanoscale range (1–100 nm), exhibit unique physicochemical properties, including enhanced mechanical and thermal stability, increased surface area, and distinctive optical, electrical, and magnetic behaviors. These properties arise primarily from quantum confinement effects and the high surface-to-volume ratio characteristic of nanoscale systems [48]. These characteristics render nanomaterials relevant for applications in medicine, agriculture, and environmental metal technologies [49]. Additionally, their tunable surface chemistry enables functionalization for targeted and application-specific performance [50]. Nanomaterials can be classified according to their composition into three main categories (Fig. 2): carbon-based, inorganic, and organic [51]. Carbon-based materials include hollow spheres, ellipsoids, or tubes, exemplified by fullerenes, nanodiamonds, and graphene [52]. These materials are particularly valued for their exceptional mechanical strength, electrical conductivity, and chemical stability [53]. Inorganic nanomaterials encompass metallic nanoparticles such as silver (Ag), gold (Au), and iron (Fe), as well as metal oxides, including zinc oxide (ZnO) and cerium oxide (CeO_2_), which present optical and electrical properties at the nanoescale [54]. Such properties make them especially suitable for catalysis, sensing, and antimicrobial applications [55]. Organic nanomaterials consist mainly of lipids, polysaccharides, or polymers, typically ranging from 10 nm to 1 μm [56]. They are widely explored in drug delivery systems due to their biocompatibility, biodegradability, and low toxicity [57].

Fungi have emerged as efficient biological agents for the biosynthesis of nanomaterials due to their enzymatic systems, metal-reducing capabilities, stability of agglomerates, and ability to tolerate and interact with toxic metals as lead (Pb), copper (Cu), and cadmium (Cd) [58, 59]. This tolerance is largely attributed to adaptive cellular mechanisms, including metal sequestration, efflux systems, and intracellular detoxification pathways [60, 61]. Among metallic nanoparticles, silver, gold, and zinc nanoparticles have been investigated due to their physicochemical properties and biotechnological applications. These nanoparticles exhibit distinct functionalities, such as antimicrobial activity (Ag) [62], catalytic and optical properties (Au) [63], and photocatalytic and semiconducting behavior (Zn) [64]. Fungal-mediated synthesis of these nanoparticles utilizes specific metal precursors, such as silver nitrate (AgNO_3_), chloroauric acid (HAuCl_4_), and zinc salts. During this process, fungal biomolecules act both as reducing agents, converting metal ions into their elemental form, and as capping agents, ensuring nanoparticle stability and preventing aggregation. Furthermore, the use of fungal systems enables synthesis under mild conditions, reducing energy requirements and minimizing the generation of hazardous by-products compared to conventional methods.

#### Predominant metallic nanoparticles: silver, gold, and zinc

Silver, gold, and zinc nanoparticles synthesized via fungal methods exhibit size and morphological variations that depend on the specific procedures employed and the fungal strains used (Table S3). These variations are influenced by factors such as pH, temperature, precursor concentration, and incubation time, which directly affect nucleation and growth mechanisms. Each metal has distinct applications; therefore, the choice of fungal strain should be guided by the intended application. This highlights the importance of tailoring biosynthesis conditions to achieve application-specific nanoparticle characteristics. For silver, antimicrobial activity is the primary application, and a smaller particle size (< 20 nm) correlates with enhanced antimicrobial efficacy [65, 66]. This enhanced activity is mainly attributed to increased surface area and improved interaction with microbial cell membranes, leading to disruption of cellular functions. These attributes have driven their widespread use across biomedicine, food packing, textiles, agriculture, and water treatment [67, 68]. Moreover, their broad-spectrum antimicrobial activity against bacteria, fungi, and even some viruses further expands their applicability. Fungi that produce small silver nanoparticles, such as *A.sydowii (*1–24 nm) [69], *A.fumigatus* (3–13 nm) [70], and *Penicillium citrinum* (3–13 nm) [71–73] are considered valuable for this purpose. These species are particularly relevant because they consistently produce nanoparticles with a controlled size distribution and high stability.

For gold nanoparticles, their biocompatibility and inert nature have further positioned them as promising tools in biomedicine, where they are applied in protein labeling, cell identification, and imaging [74, 75]. In 2019, the U.S. Food and Drug Administration (FDA) approved CNM-Au8, a drug based on gold nanoparticles for Amyotrophic Lateral Sclerosis (ALS), underscoring the translational potential of these nanomaterials [76]. However, regulatory approval for broader therapeutic use remains limited due to safety concerns linked to particle size, morphology, and synthesis route [77]. For this purpose, a small size (< 20 nm), monodisperse gold nanoparticles are preferred, as seem in studies using *A.terreus* (9–14 nm) [78] and *Alternaria* sp. (9.5 ± 2.9 nm) [79].

Zinc-based nanomaterials have biodegradability, low cost, and functional versatility. These characteristics make them particularly attractive for sustainable and large-scale industrial applications. As an essential micronutrient, zinc participates in fundamental biological processes, including antioxidant defense through enzymes such as superoxide dismutase (SOD) [80, 81]. This biological relevance also contributes to their generally low toxicity and high biocompatibility compared to other metal-based nanomaterials. Among its nanoscale derivatives, zinc oxide nanoparticles (ZnONPs) are the most studied. Their widespread use in pigments, ceramics, cement composites, and rubber is supported by intrinsic properties such as thermal conductivity, stability, and antimicrobial activity [82]. Additionally, their semiconducting properties enable applications in photocatalysis and sensor technologies. Moreover, their ability to absorb ultraviolet radiation and resist photodegradation has made them valuable additives in sunscreens and other protective formulations [83]. These features are directly related to their wide band gap and strong UV absorption capacity. For these applications, monodisperse particles in the 10–70 nm range, such as those produced by *A.niger* [84] and *A.terreus* [85], balance UV protection efficacy with controlled dermal penetration [84]. The monodispersed of *Cordyceps militarir (*7–16 nm) [86] makes this species particularly promising for biomedical applications of zinc nanoparticles. Such precise size control is essential for drug delivery, imaging, and other nanomedicine applications, where particle behavior is highly size-dependent.

#### Size diversity and the influence of methodology

Variability in size is a prominent pattern observed across all three metals and may occur within the same fungal species. For silver, *Aspergillus fumigatus* has been reported to produce particles ranging from 3 to 13 nm ([70], 17–75 nm [87] and 49.0 ± 19.6 nm [88]. Similarly, *Aspergillus niger* yields silver nanoparticles as small as 3–13 nm [89] or as large as 93.3 ± 28.3 nm [88].

For gold, *Botrytis cinerea* produces particles of 92.9 ± 231, while *Alternaria* sp. and *A.terreus* produce smaller, more monodisperse particles at 9.5 ± 2.9 nm and 9–14 nm, respectively [78, 79]. For zinc, *Phanerochaete chrysosporioum* yields particles spanning 5–200 nm [90], reflecting minimal kinetic control over nucleation, whereas *Cordyceps militaris* produces monodisperse particles at 7–16 nm [86].

This variability cannot be attributed solely to biotic factors; rather, it indicates a lack of methodological standardization across laboratories. Parameters such as temperature, pH, incubation duration, fungal biomass concentration, precursor concentration, and agitation conditions vary significantly across studies. Among the 100 articles reviewed, 50% did not report any statistical quality assessment, and of those that did, only 10% included a control group. This omission is not a minor oversight; it directly limits the utility of the published data. Without statistical descriptors, size differences within the same species, such as *A. fumigatus*, producing both 3–13 nm [70] and 75 nm [87] Silver nanoparticles cannot be meaningfully categorized.

A key methodological discrepancy in nanoparticle synthesis concerns their purification processes. Most protocols employ centrifugation to separate nanoparticles; traditionally, this method is viewed solely as a technique that exploits density differences among particles. However, centrifugation also acts as an active factor that can induce predictable morphological transformations [91]. While higher centrifugation speeds (e.g., 20,000 rpm) can sediment particles smaller than 20 nm, this does not necessarily imply that higher speeds are optimal for all nanoparticle types, as increased speeds may promote aggregation due to intensified collision forces [92].

Therefore, determining the appropriate centrifugation speed presents a significant challenge; reported conditions exhibit considerable variability, ranging from 5,000 to 20,000 rpm [93, 94] and from 5,000 × g to 14,000 × g [95, 96]. An additional complication arises from insufficient reporting of centrifugation parameters in the literature, such as temperature, loading volume, and centrifuge tube type. Moreover, many reports focus solely on centrifugation speed expressed in rpm, neglecting the relative centrifugal force (g), which complicates protocol standardization [97].

To remove excess solvent or water and obtain a final dry powder, nanoparticles undergo a drying process that can also influence their morphology. The most commonly employed method is oven drying [73, 90, 95, 98, 99], due to its simplicity, conventionality, and cost-effectiveness, followed by hot-air oven drying [100, 101]. Some studies have utilized lyophilization (freeze-drying) [69, 86, 102]. This step, however, can potentially compromise the integrity of the nanoparticles. Oven drying may lead to nanoparticle agglomeration and structural damage. The underlying mechanism involves water evaporation, which generates surface tension that physically pulls particles together, reduces interlayer spacing, and can result in structural collapse [103]. In contrast, lyophilization (freeze-drying) is considered a superior technique because it preserves the original nanostructure by sublimating ice directly to vapor under vacuum, thereby avoiding capillary forces and surface-tension effects that cause agglomeration during conventional drying [104]. Nonetheless, due to its longer processing time, higher cost, and the presence of solvents such as alcohol used in nanoparticle synthesis, lyophilization may be an invaluable method for certain applications. L.

#### Fungal cultivation parameters affecting biosynthesis

Among the studies reviewed, Potato Dextrose Broth (PDB) was the most frequently utilized medium, accounting for 44% of the cases. Defined media were employed in 15% of the studies, followed by Malt Glucose Yeast Peptone (MGYP) at 9%, Sabouraud agar at 6%, malt extract at 5%, and Czapek-Dox broth at 4%. The remaining 17% of studies used other or unspecified media. The choice of culture medium affects fungal growth and the biosynthesis of bioactive compounds, including secondary metabolites involved in the reduction and stabilization of nanoparticles [105]. Fungi adjust their metabolic pathways in response to environmental conditions; thus, an optimal medium must support both mycelial growth and the activation of biosynthetic gene clusters (BGCs). Liquid culture systems predominate because they generate cell-free filtrates enriched in bioactive metabolites. PDB is favored for its simplicity, cost-effectiveness, and broad compatibility with genera such as *Ganoderma* (Basidiomycota) [79], *Mortierella* (Mortierellomycota) [106], *Rhizopus* (Mucoromycota) [93], and *Tricholoma* (Basidiomycota) [107].

Defined control nutrient composition, enabling reproducible metabolite biosynthesis and nanoparticle morphology evaluation. For example, *Trichoderma longibrachiatum* cultured in defined media produced monodispersed spherical silver nanoparticles (~ 10 nm) stable for over two months, which reduced 90% of phytopathogenic fungi [108]. The medium Malt Glucose Yeast Peptone (MGYP) favors the production of secondary metabolites and assists in the biosynthesis of nanoparticles. In *Humicola* sp [109]., α-Ag2S nanoparticles (~ 20 nm) exhibited antimicrobial and cytotoxic activity. Nevertheless, systematic studies directly comparing the influence of different media on fungal growth and secondary metabolites mediated by nanoparticle biosynthesis remain scarce. For instance, *Fusarium solani* yielded higher biomass in Sabouraud Dextrose Broth (SDB) compared with Czapek dox (CDB) and PDB [110], likely due to the availability of fast-assimilating carbon and nitrogen sources. Consequently, a critical research gap exists: the comparative effectiveness of nanoparticle biosynthesis across media remains underexplored. Transitioning from complex to defined media represents a strategic step toward process standardization, enhanced nanoparticle quality, and scalable production. Future studies should integrate medium optimization with assessments of nanoparticle yield, morphology, and stability.

The reporting of pH conditions was notably insufficient. Only 21% of the studies specified the pH used, with pH 5 at 6%, pH 6 at 6%, pH 7 at 5%, and pH 9 at 4%. The remaining 79% failed to report the optimal pH for nanoparticle synthesis. Furthermore, none of the studies within the reported 21% provided information regarding the initial pH. This parameter influences nanoparticle formation and colloidal stability. Most studies focus on silver nanoparticles, with less attention to other metals. Alkaline conditions increased silver nanoparticle formation by *Arthroderma fulvum* [111], while *Colletotrichum siamense* showed optimal synthesis at pH 8.77 [112]. In *Guinardia magallanicae*, synthesis was inhibited at pH 3 and 4, whereas pH 7 produced monodisperse and stable nanoparticles [113]. Alkaline conditions facilitate rapid reduction of metal ions, as hydroxyl ions enhance Ag+ reduction and reduce competition at negatively charged binding sites. For instance, *Penicillium chrysogenum* synthesized stable silver nanoparticles within 12 h at pH 11 [114]. Conversely, acidic conditions can limit enzyme activity, reducing nanoparticle formation.

Not all metals respond similarly. In gold nanoparticle biosynthesis using *Penicillium rubens*, acidic environments favored formation due to protonation effects on functional groups involved in biomolecular reduction [115]. Similarly, *Tricholoma crassum* produced distinct gold nanoparticles characteristic of varying pH, highlighting the importance of colloidal stability and enzyme activity in process optimization [107]. Considering nanoparticle yield and stability, pH must be carefully optimized for each microbial-metal system. Broadening these studies beyond silver nanoparticles uncovers efficient, metal-specific biosynthetic routes.

The most cited mycelial growth times for fungi are 5 days (120 h, 22%), 3 days (72 h, 18%), and 4 days (96 h, 12%). Filamentous fungi can adapt well to different environmental conditions, and the length of cultivation significantly affects their development and metabolic functions. The literature indicates that harvesting typically occurs between 3 and 5 days (72–120 h), during the late exponential to early stationary phases, when extracellular biomolecules are most abundant [93, 114, 116–119]. For example, *Mucor circinelloides* [120] yielded up to 28.1% of antioxidant biomolecules at 5 days, higher than at 3 days, whereas no difference was observed between 5- and 7-day cultures. Mycelial morphology, ranging from dispersed hyphae to compact pellets, also affects metabolite output. However, few studies systematically account for morphological variability during growth [121, 122]. Thus, integrating precise growth-phase monitoring and morphological assessment is essential to optimize the reproducibility of nanoparticle biosynthesis and process efficiency. Future research should couple metabolic profiling with dynamic growth analyses to identify optimal harvesting windows.

Although PDB was the most frequently used cultivation medium, its prevalence does not necessarily indicate superior performance in nanoparticle biosynthesis. Systematic comparisons of different culture media under similar experimental conditions remain limited. *Fusarium solani* produced higher biomass in Sabouraud Dextrose Broth than in Czapek-Dox broth and PDB; however, increased biomass alone does not necessarily correlate with enhanced nanoparticle production or better control over nanoparticle properties. The existing literature still lacks sufficient evidence to determine how medium composition influences nanoparticle yield, size distribution, morphology, or colloidal stability. This represents a significant methodological gap, especially since changes in nutrient composition can affect fungal growth and the production of enzymes, secondary metabolites, and other biomolecules involved in metal transformation and nanoparticle stabilization. Future studies should systematically compare cultivation media while controlling or independently varying biomass concentration, precursor concentration, pH, temperature, and incubation time. Such research should evaluate nanoparticle physicochemical outcomes, including yield, size distribution, and morphology, to establish whether medium composition has a direct and reproducible effect on fungal-mediated nanoparticle synthesis.

#### Beyond silver, gold, and zinc: underexplored metals

**Fig. 3:**
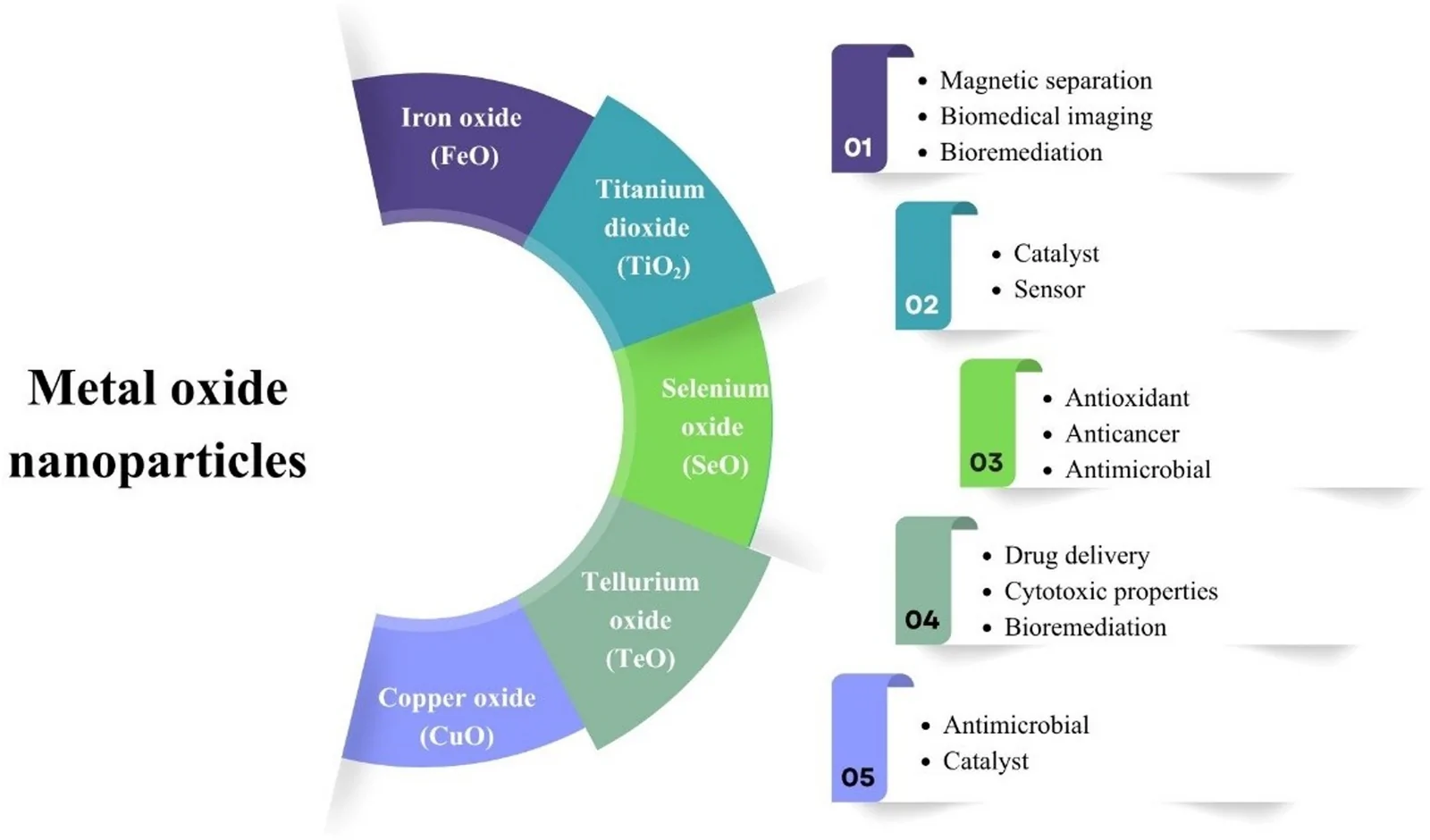
Schematic representation of metal oxide nanoparticles and their multifunctional applications

While silver, gold, and zinc are well- covered in the literature on fungal-mediated nanoparticles, fungi can produce a wider range of metallic, metal oxide, and metalloid nanoparticles (Fig. 3). These include, for instance, iron oxide, titanium dioxide, selenium, tellurium, and copper-based nanostructures, each exhibiting distinct physicochemical properties and potential applications [123]. These lesser- studied systems offer unique properties, such as magnetic, catalytic, photocatalytic, or semiconducting features, that could greatly broaden the applications of fungal nanotechnology. For example, iron oxide nanoparticles enable magnetic separation and targeted drug delivery, while titanium dioxide and zinc-based composites are widely explored for photocatalytic degradation of pollutants [124–126].

Despite their promising functionalities, these alternative nanomaterials remain underexplored due to challenges in controlled synthesis, reproducibility, and a limited mechanistic understanding of fungal–metal interactions. Differences in cell wall composition, secreted metabolite profiles, and enzymatic systems across fungal species can significantly influence reduction kinetics, nucleation pathways, and final nanoparticle characteristics [10]. Advancing research in this area requires integrative approaches combining metabolomics, proteomics, and genomics to elucidate the molecular basis of metal transformation and nanoparticle stabilization. Such insights would enable the rational selection or engineering of fungal strains tailored to synthesize specific nanomaterials with desired functionalities. Consequently, expanding the focus beyond conventional metals represents a critical step toward unlocking the full biotechnological potential of fungal nanoplatforms and enabling next-generation applications in environmental remediation, energy systems, and nanomedicine.

Magnetic iron or iron oxide nanoparticles are especially valuable for environmental cleanup, magnetic separation, and biomedical imaging [127]. optimized producing magnetic nano- iron with *Aspergillus flavipes* used for dye decolorization and antifungal effects [128]. Bedi et al. (2018) described *Aspergillus aculeatus* strains capable of creating agriculturally useful nanoparticles via bioremediation of iron tailings [129]. biofabricated iron oxide nanoparticles using mangrove fungus *Aspergillus niger* and used them to remove Cr(VI) from water.

Copper-based nanoparticles exhibit strong antimicrobial and catalytic activities [130]. created copper nanoparticles that inhibit the growth of plant pathogens like *Fusarium oxysporum* and *Phytophthora parasitica* [131]. produced copper carbonate nanoparticles with *Neurospora crassa* fungi, revealing interactions between amino acids and copper minerals [132]. showed that *Aspergillus terreus* can produce silver- copper bimetallic nanoparticles.

Selenium nanoparticles attract interest for their antioxidant, anticancer, and antimicrobial properties with low human toxicity. *Penicillium citrinum* and *Rhizopus arrhizus* can synthesize selenium nanoparticles approximately 50–80 nm in size. *Penicillium exapandum* [133] also demonstrated biomedical potential for selenium nanoparticles in antioxidant, antimicrobial, and anticancer applications.

The green synthesis of nanoparticles from critical metals, such as niobium (Nb), hafnium (Hf), zirconium (Zr), and vanadium (V), presents different challenges than those encountered for noble metals (E°(Ag^+^/Ag^0^) = + 0.80 V;, E°(Au^3+^/Au^0^) = + 1.50 V) [134, 135]. Whereas the high positive reduction potentials of Ag^+^ and Au^3+^ enable straightforward enzymatic reduction to the metallic state, the highly negative reduction potentials of Nb (E°=-1.10 V), Hf (E° = -1.55 V), Zr (-1.53 V), and V (E° = -1.18 V) [135] preclude analogous biological reduction pathways. Instead, as demonstrated by Hamzah in the synthesis of V_2_O_3_ nanoparticles using *Fusarium solani*, these refractory metals undergo partial reduction coupled with hydrolysis, yielding stable oxide nanoparticles as the final products [136]. In the case of zirconium, *Penicillium purpurogenum*, *Penicillium notatum*, and *Penicillium aculeatum* have successfully synthesized nanoparticles of 14. 84 nm, 14.14 nm, and 12.55 nm, respectively [137]. Similarly, *Aspergillus flavus* was the first fungus reported to synthesize Nb_2_O_5_ nanoparticles [138].

These findings indicate that, despite thermodynamic constraints and limitations, certain fungal strains possess metabolic pathways capable of mediating the controlled nucleation and growth of nanoparticles within a relatively narrow size distribution; the biomolecules can serve. Biomolecules such as proteins, polysaccharides, and phenolic compounds can act as effective capping and anti-agglomeration agents, stabilizing particles despite incomplete reduction [139, 140]. These biomolecules interact with metal ions and partially reduced species through coordination bonds and steric hindrance, leading to the formation of stable nanostructures [141]. Additionally, mechanisms such as biomineralization and enzyme-mediated redox cycling may contribute to nanoparticle formation when full reduction to the zero-valent state is not achieved. This suggests that fungal systems can facilitate the synthesis of metal oxides or hybrid nanostructures, thereby broadening the range of obtainable nanomaterials beyond purely metallic nanoparticles [91].

Despite these encouraging examples, research on these less-explored metals is still in its early stages. Future work should focus on understanding mechanisms, optimizing processes, and scaling up production for these systems.

#### Morphological patterns and functional performance

Across all three metals, spherical or quasi-spherical nanoparticles predominate. This convergence reflects thermodynamic favorability, as spherical particles represent the lowest energy configuration during nucleation and growth [142, 143]. However, the exceptions to this pattern are more informative than the rule, revealing when kinetic factors override thermodynamic preferences. For silver, hexagonal particles have been reported from *Emirecella nidulans* [144]. For gold, *Fusarium solani* produces needle-like and flower-like structures [145], while *Tricholoma crissum* generates rhomboid, hexagonal, and cuboidal morphologies [107]. For zinc, *Aspergillus fumigatus* produces flower-like zinc phosphate nanosheets [146], and *Phanerochaete chrysosporium* yields hexagonal particles [90]. These atypical morphologies suggest species-specific metabolic pathways that may involve proteins with directional roles in crystal growth, overriding the default spherical outcome. Deliberately targeting fungi known to produce such exceptions could unlock mechanistic insights applicable to tailoring nanoparticle shape for specific applications, such as biomedical, catalytic, or environmental applications. However, morphology should not be considered independently of particle size and fungal-derived surface capping, as these characteristics collectively influence surface accessibility, colloidal behavior, and interactions with biological or environmental systems. Representative physicochemical characteristics and the corresponding functional performance of fungal-synthesized nanoparticles are summarized in Table 2.

**Table 2:** Representative Physicochemical Characteristics and Functional Applications of Fungal-Synthesized Nanoparticles

Nanoparticle/Fungal system	Physicochemical characteristic	Functional performance	Reference
Silver nanoparticles/*Trichoderma longibrachiatum*	~ 10 nm spherical and monodispersed; colloidally stable for > 2 months	Strong antifungal activity (90%) against plant pathogens	[108]
Silver nanoparticles/ *Aspergillus fumigatus*	0.0681 nm cube-shaped, monodispersed particles	Antibacterial activity against multidrug-resistant bacterial strains; enhanced antiproliferative and cytotoxic effects when combined with cisplatin	[147]
Gold nanoparticles/Tricholoma crassum	5–25 nm; different geometries; naturally protein-capped	Antimicrobial activity against bacteria, fungi, and multi-drug-resistant bacteria	[107]
Gold nanoparticles/*Fusarium solani*	Average diameter ~ 40 and 45 nm	Cytotoxicity and cell lysis	[145]
Zinc nanoparticles/*Trichoderma harzianum*	8–23 nm; rod and hexagonal morphologies	Fungicidal action against soil-cotton pathogenic fungi	[148]
Zinc nanoparticles/*Cordyceps militaris*	1.83 nm spherical to oval shape particles; moderately stable zeta potential	Antidiabetic, antioxidant, and antibacterial potential	[86]

### Characterization techniques

**Fig. 4:**
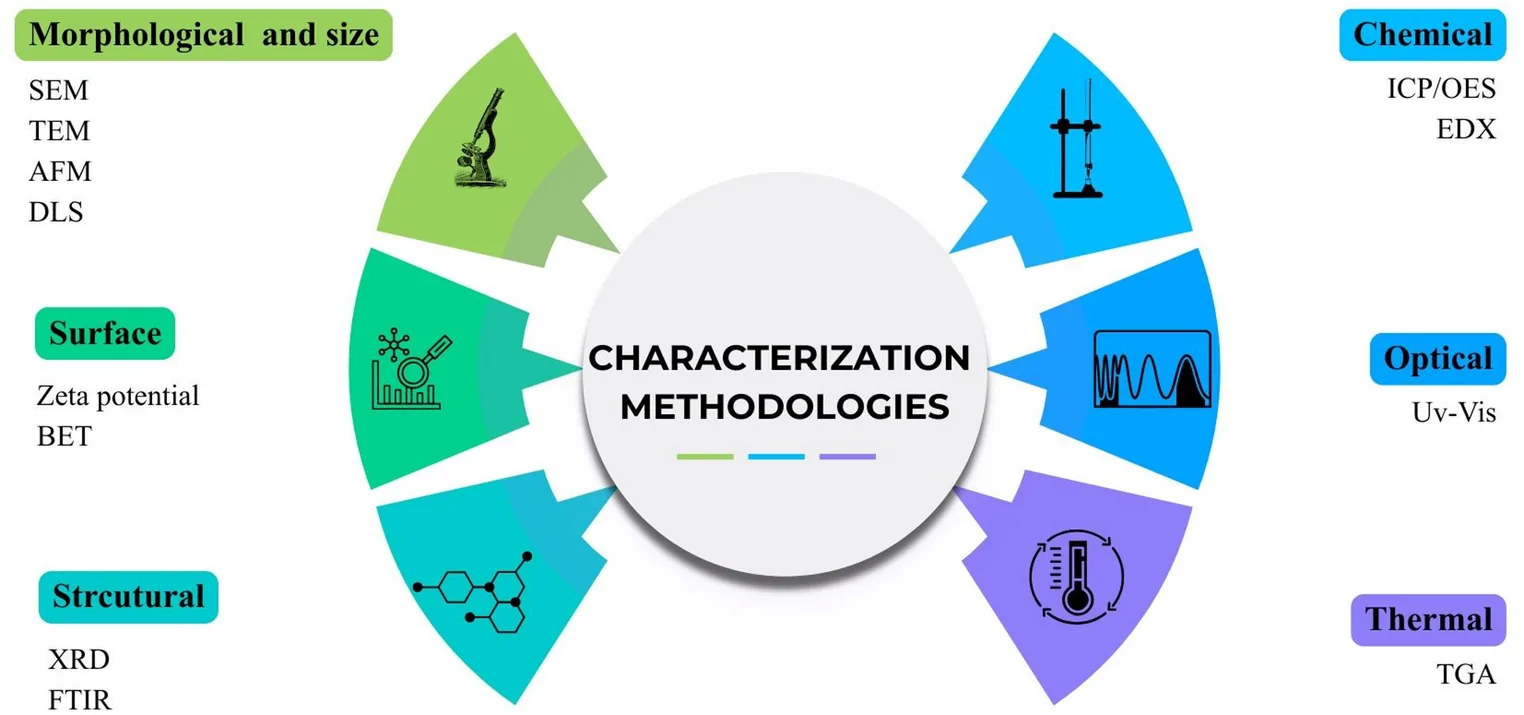
Main characterization methodologies for nanoparticles

Nanoparticles synthesized via green methodologies using microorganisms or plants require comprehensive characterization with a range of techniques to assess their properties, including size, shape, crystallinity, surface chemistry, and elemental composition (Fig. 4). Each characterization method is selected based on the specific property being examined. Thorough analysis of these nanoparticles is crucial for unlocking their full functional potential while ensuring their safety and efficacy. Understanding the physicochemical properties and behaviors of nanoparticles enables researchers to make evidence-based decisions, advance technological development, and promote the ethical and sustainable progression of nanotechnology [149].

#### Morphological and size analysis

Microscopy represented a significant advancement, enabling the observation of materials at the micron scale. Nevertheless, it is limited by factors such as the light’s wavelength and lens aberrations. To surpass these restrictions and analyze features smaller than a micron, researchers developed more advanced imaging techniques, including Atomic Force Microscopy (AFM), Scanning Electron Microscopy (SEM), and Transmission Electron Microscopy (TEM). While each technique is based on different operational principles, they all produce magnified images capable of revealing nanoparticles.

Scanning Electron Microscopy is appropriate for analyzing the surface topography of dry, conductive, or coated nanoparticle specimens, achieving a spatial resolution of approximately 1–5 nm. When combined with Energy-Dispersive X-ray (EDX) spectroscopy, SEM facilitates elemental composition mapping, which is particularly valuable for identifying metal-based nanomaterials such as gold, silver, or niobium. For example, SEM-EDX was used to verify that the Strontium Oxide (SrO) particles were synthesized by *Aspergillus* sp. LCJ315 was spherical and contained 25.1% Sr by weight [150].

Transmission Electron Microscopy (TEM) and its high-resolution variant, High-Resolution Transmission Electron Microscopy (HRTEM), enable visualization of internal crystallographic structures, crystal defects, and even individual atoms, provided the specimen is prepared as an ultrathin foil. In fungal-mediated nanoparticles, TEM is frequently used to determine their size and shape. For example, TEM analysis revealed that silver nanoparticles biosynthesized by *Colletotrichum incarnatum* are well dispersed, spherical, and range in size from 5 to 25 nm [151]. Similarly, Aspergillus fumigatus produced spherical nanoparticles measuring 17.11–75.54 nm, as observed by HRTEM [119].

For three-dimensional surface profiling without the need for a conductive coating, Atomic Force Microscopy employs a sharp cantilever tip to scan the sample surface, measuring topography with angstrom-scale vertical resolution while preserving sample integrity. AFM was effectively utilized to characterize gold nanoparticles synthesized by *Tricholoma crassum*. The analysis revealed that the surface heights of the nanoparticles ranged from 1 to 4 nm. Additionally, the surface plasmon resonance band suggested that the particle thickness was less than its lateral dimensions [107].

Although all these techniques produce highly magnified images, they are often complemented by Dynamic Light Scattering (DLS), which rapidly assesses the average hydrodynamic diameter and size distribution of nanoparticles in suspension, providing statistical data that enhances the localized, detailed insights obtained from microscopy. In fungal nanoparticles biosynthesis, DLS is frequently employed alongside microscopy to verify nanoparticle stability and monodispersity [152, 153]. For example, DLS analysis confirmed that the biosynthesized silica nanoparticles produced by *Aspergillus niger* had an average particle size of approximately 70.1 nm. This finding is consistent with TEM observations, which revealed spherical nanoparticles ranging from 75 to 115 nm in size [102].

#### Surface, structural, and optical analysis

Additional techniques are necessary to assess the chemical composition, crystalline nature, surface functionality, and porosity of nanomaterials. X-ray diffraction (XRD) is a nondestructive technique used to determine the crystallinity, phase composition, and crystallite size in nanoparticles. When an X-ray beam encounters a crystalline sample, it is diffracted at specific angles that correspond to the spacing between atomic planes, in accordance with Bragg’s law [154, 155]. The resulting diffraction pattern serves as a fingerprint of the crystal structure, enabling identification of the material’s phase (such as cubic, tetragonal, or hexagonal) and any impurities present. This analysis provides essential insights into how crystallinity influences properties like thermal stability, optical behavior, and catalytic activity [156]. Additionally, peak broadening in the diffraction pattern can be used to estimate the average crystallite size via the Scherrer equation; although this correlates with sizes observed through transmission electron microscopy (TEM), it does not replace direct imaging. In fungal-mediated synthesis, XRD has been employed to confirm the formation of metallic silver or gold nanoparticles with face-centered cubic (FCC) crystal structures [157–159].

Fourier Transform Infrared Spectroscopy (FTIR) is employed to identify surface functional groups on nanoparticles and to elucidate the chemical interactions between metal ions and capping biomolecules derived from biological synthesis methods, such as fungal or plant extracts. This technique functions by transmitting infrared radiation through a sample and recording the absorption at different wavenumbers, with each functional group producing a characteristic absorption band [160, 161]. Studies on fungi-mediated nanoparticles consistently reveal several characteristic absorption bands, indicating the involvement of specific biomolecular classes. A broad, intense band between 3200 and 3450 cm^− 1^ overlaps O-H and N-H stretching vibrations, originating from hydroxyl groups in alcohols, phenols, or polysaccharides, as well as from amide groups of fungal proteins. For example, in AgO nanoparticles, a band at 3441.01 cm^− 1^ indicated hydroxyl groups from alcohol-class compounds [162]. Similarly, a band at 3351 cm^− 1^ in fungal biomass and another at 1663 cm^− 1^ in purified silver nanoparticles suggest metabolite-driven stabilization [163]. Amide I (1600–1650 cm^− 1^) and Amide II (1510–1550 cm^− 1^) bands confirm protein capping via C = O stretching and N-H bending, respectively [157]. Aliphatic C-H stretches between 2850 and 2960 cm^− 1^ originate from methyl and methylene groups of fungal metabolites [164]. Additionally, C-N and C-O stretches between 100 and 1250 cm-1 indicate the presence of amine and carboxylate groups that bind metal ions. A reduction in peak intensity or a shift in these peaks relative to the crude fungal filtrate indicates active consumption of biomolecules during synthesis. Low-wavenumber bands below 700 cm-1 confirm metal-oxygen or metal-sulfur bond formation [165]. Overall, FTIR analysis indicates that fungal proteins, polysaccharides, and phenolics serve as dual reducing and capping agents in nanoparticle synthesis.

Zeta potential (ζ) serves as an indicator of the surface charge of a nanomaterial and offers initial insights into its colloidal stability. When a nanomaterial is dispersed within an ionic solution, the charged surface particle is encased by an electrical double layer (EDL). The innermost part of this layer, known as the Stern layer, consists of ions that are bound directly to the particle surface. Adjacent to the Stern layer is the diffuse layer, which contains loosely associated counterions extending outward to the slipping plane, the hypothetical boundary separating the moving liquid layer from the bulk solution. The zeta potential is defined as the electrical potential at this slipping plane [166]. The magnitude of zeta potential directly influences the electrostatic stabilization of colloids; values greater than 30 mV, ideally above 60 mV, generate sufficient interparticle repulsion to counteract van der Waals attractive forces, thereby preventing aggregation and ensuring colloidal stability [167]. Conversely, values between 5 and 15 mV indicate incipient flocculation, while those below 5 mV lead to rapid aggregation and sedimentation. In the context of fungal-mediated synthesis, the predominance of negatively charged carboxylate groups derived from fungal proteins is due to the ionizable nature of these functional groups. This imparts a negative zeta potential to the nanoparticles, which promotes electrostatic repulsion and contributes to their long-term colloidal stability [144, 168, 169].

The Brunauer-Emmett-Teller (BET) theory explains how gas molecules physically adsorb onto solid surfaces and is widely used to determine the specific surface area of powdered nanomaterials, including those mediated by fungi. This technique involves measuring the volume of an inert gas, usually nitrogen, adsorbed onto the sample at 77 K over a range of relative pressures. Data collected at relative pressures between 0.05 and 0.3 are used to calculate the monolayer capacity, which then yields the specific surface area in square meters per gram. A high surface area, typically between 50 and 150 m²/g, depending on the metal, indicates well-dispersed particles with increased reactivity and more active sites, which are advantageous for catalytic, biomedical, or environmental applications. Conversely, a low surface area (< 10 m²/g) may indicate significant particle aggregation, poor dispersion, or the presence of thick biomolecular capping layers, such as polysaccharides or proteins in fungal-mediated nanoparticles, that physically hinder gas adsorption [165, 170, 171].

UV-Vis spectroscopy is a rapid and straightforward technique for characterizing nanoparticles by measuring their light absorption over the wavelength range 180–780 nm. When specific chemical groups known as chromophores absorb radiation, electrons transition from ground to excited states, typically involving σ, π, or n orbitals to antibonding orbitals. Unlike atomic spectra, molecular UV-Vis spectra exhibit broad absorption bands, which result from electronic transitions accompanied by vibrational and rotational transitions that cannot be individually resolved by conventional spectrophotometers [172]. In the case of metal nanoparticles, the most significant phenomenon is surface plasmon resonance (SPR), which occurs when incident light induces collective oscillations of conduction electrons at the nanoparticle surface. The position, intensity, and width of the SPR peak depend on various factors, including nanoparticle size, shape, composition, aggregation state, and the local dielectric environment [149]. For example, silver nanoparticles typically display a characteristic SPR peak between 420 and 500 nm; smaller, well-dispersed particles produce sharper and more intense bands, whereas aggregation results in peak broadening and a redshift [173]. Consequently, UV-Vis spectroscopy is an efficient method for confirming nanoparticle formation and evaluating colloidal stability.

#### Chemical and thermal analysis

Inductively Coupled Plasma Optical Emission Spectroscopy (ICP-OES) is a precise, multi-element analytical technique used to quantify metal concentrations in nanoparticles. It offers high sensitivity with low detection limits, ranging from milligrams per liter to micrograms per liter. This method is valuable for determining nanoparticle yield, evaluating purification efficiency, monitoring colloidal stability over time, and quantifying metal content. When integrated with other analytical techniques such as Scanning Electron Microscopy (SEM), Transmission Electron Microscopy (TEM), Fourier-Transform Infrared Spectroscopy (FTIR), and X-ray Diffraction (XRD), ICP-OES provides essential quantitative data that complement the physicochemical characterization of fungal-mediated nanoparticles [174, 175].

Thermogravimetric analysis (TGA) monitors changes in sample mass as a function of temperature under controlled atmospheric conditions, offering essential insights into the thermal stability, composition, and decomposition patterns of nanoparticles and their organic layers. Typically, a small sample ranging from 2 to 10 mg is heated at a steady rate, usually 10 °C per minute, while a high-precision balance records weight changes continuously. The resulting thermogram plots the remaining mass percentage against temperature [176, 177]. The derivative thermogravimetric (DTG) curve highlights specific temperatures where the most rapid weight loss occurs, pinpointing key thermal events. In the case of nanoparticles synthesized via fungal mediation, TGA is particularly useful for quantifying biomolecular capping agents, such as proteins, polysaccharides, and secondary metabolites, adsorbed on the particle surface. These biomolecules directly impact colloidal stability and biological activity [73, 90].

Characterization of fungal-mediated nanoparticles presents additional challenges because the resulting materials are commonly associated with proteins, polysaccharides, metabolites, and other extracellular components derived from the fungal systems [178]. Although techniques such as FTIR can indicate the presence of functional groups with biological capping, they do not determine the composition or abundance of individual molecules forming the biomolecular corona [179]. Complementary approaches are therefore required to distinguish surface-bound biomolecules from residual biological impurities remaining after synthesis and purification, as these components may interfere with spectroscopic measurements and influence particle size, surface charge, and biological activity. TGA can provide an estimate of the overall organic fraction, whereas more detailed chemical or molecular analyses are necessary when the identity of individual corona components is relevant. Colloidal stability should also be evaluated under application-relevant conditions, as variations in pH, ionic strength, and medium composition can alter nanoparticle aggregation and surface properties [180]. Together, these considerations highlight the need for complementary characterization approaches capable of distinguishing intrinsic nanoparticle properties from effects associated with the surrounding biological matrix.

#### Recommended analytical method combination

Comprehensive nanoparticle characterization requires complementary analytical methods, as individual techniques assess different physicochemical properties and may not independently provide sufficient evidence of nanoparticle identity or behavior. The appropriate analytical combination should therefore be selected according to the nanoparticle composition, physical state, and intended application. Table 3 summarizes the principal nanoparticle properties and the corresponding complementary analytical approaches recommended for their characterization.

**Table 3:** Recommended analytical methods for the characterization of biologically synthesized nanoparticles

Nanoparticle property	Primary analytical method(s)	Complementary method(s)
Nanoparticle formation/optical behavior	UV-Vis absorption spectroscopy for colloidal suspensions; UV-Vis diffuse reflectance spectroscopy (DRS) for solid samples	TEM/SEM, XRD
Particle size, morphology, and size distribution	TEM and/or SEM	DLS; AFM when available
Crystallinity and phase composition	XRD	HRTEM when available
Elemental composition	EDX/EDS	ICP-OES, ICP-MS, or AAS
Elemental composition and metal content	EDX; ICP-OES, ICP-MS, or AAS	-
Surface chemistry and biological capping	FTIR	Raman spectroscopy
Surface change and colloidal stability	Zeta potential	DLS; UV-VIS
Specific surface area and porosity	BET	SEM/TEM
Thermal stability and organic fraction	TGA/DTG	FTIR when chemical characterization of the organic coating is required

### The mechanisms of nanoparticle biosynthesis

The ability of fungi to synthesize nanoparticles is fundamentally linked to their complex metabolic processes. Fungi produce a wide variety of chemically diverse compounds known as secondary metabolites (SMs), which play key roles in ecological interactions. Many of these compounds have been utilized by humans for technological and therapeutic applications. These secondary metabolites can be both harmful, such as mycotoxins, causing outbreaks in turkeys [181], and beneficial, exemplified by the discovery of penicillin [182]. Their biochemical relevance to nanoparticle synthesis stems from three measurable properties: redox potential (electron-donating capacity), metal-binding functional groups (hydroxyl, carboxyl, amine, carboxylate), and secretion kinetics. However, a significant gap in current research is the lack of direct molecular evidence linking specific metabolites or enzymes to nanoparticle formation. Most studies infer mechanisms from functional group presence rather than demonstrating them through molecular identification.

**Fig. 5:**
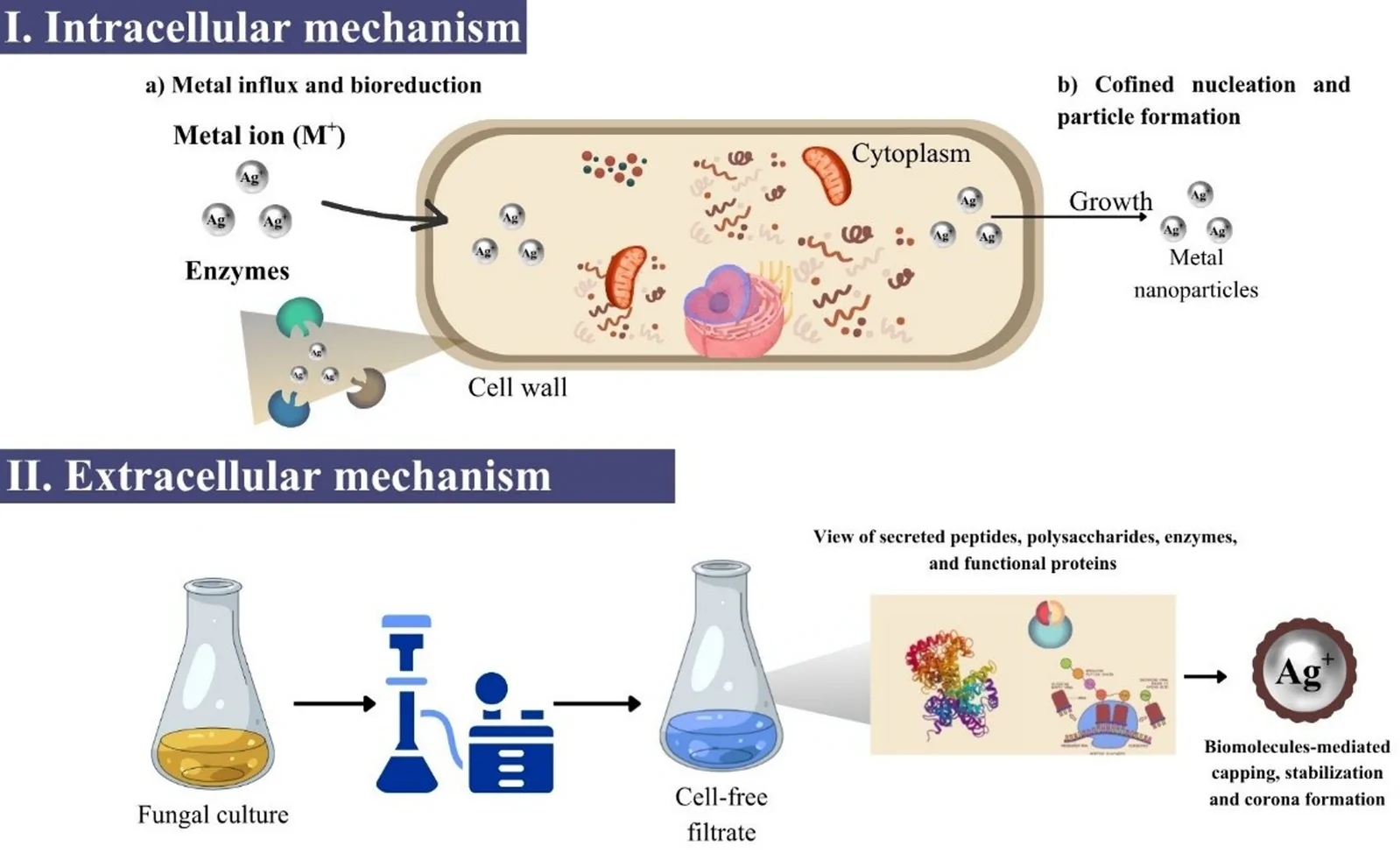
Schematic representation of intracellular and extracellular mechanisms of fungal nanoparticle synthesis

The literature identifies two primary methods for fungal nanoparticle synthesis: intracellular and extracellular pathways (Fig. 5). These methods differ in the processes of metal ion encapsulation, reduction, nucleation, and stabilization. The intracellular pathway begins when metal ions diffuse through or bind to the fungal cell wall, a biopolymeric matrix rich in negatively charged functional groups, such as carboxyl and hydroxyl groups, that can coordinate metal ions [26, 60]. This electrostatic attraction concentrates metal ions on the cell surface, facilitating subsequent reduction. Following initial binding, metal ions are transported into the cytoplasm via active or passive transport systems or remain associated with the cell wall interfacial matrix, where reduction and nucleation of nanoparticles take place [183, 184].

Once internalized, enzymatic reduction occurs. Fungi utilize intracellular enzymes like reduced nicotinamide adenine dinucleotide (NADH-) and reduced nicotinamide adenine dinucleotide phosphate (NADPH) reductases, nitrate reductases, and other oxidoreductases to transfer electrons to metal ions such as Ag^+^ and Au^3+^, reducing them to their elemental or oxide forms. Following reduction, the atoms cluster into nanostructures and develop into stable nanoparticles. Nucleation and growth happen at specific sites, including the cytoplasm, cell wall, or cell membrane, depending on the fungal species and the metal involved [141, 185]. To prevent aggregation and maintain colloidal stability, capping agents bind to the nanoparticle surface. Proteins, peptides, polysaccharides, and other metabolites serve as these stabilizing agents. Some metabolites, such as naphthoquinones and anthraquinones, also function as electron shuttles, facilitating further reduction [186].

Since nanoparticles remain trapped within fungal biomass after synthesis, extraction is a crucial downstream step that involves cell disruption, separation, and purification. The process begins with cell lysis, which can be achieved through mechanical methods such as sonication, ultrasonication, mechanical stirring, and freeze-thaw cycles; chemical methods involving solvents, surfactants, ionic liquids, and enzymatic lysis may also be employed [187, 188]. Following cell disruption, nanoparticles are separated from the biomass via centrifugation and filtration, often requiring multiple washing cycles to eliminate residual fungal material. Additional purification techniques, including gel filtration, ultracentrifugation, membrane filtration, and ion-exchange chromatography, may be utilized depending on the specific type of nanoparticle and its intended application [189, 190]. These procedures make intracellular synthesis more time-consuming and labor-intensive, and potentially less efficient in yield than extracellular synthesis methods.

**Fig. 6:**
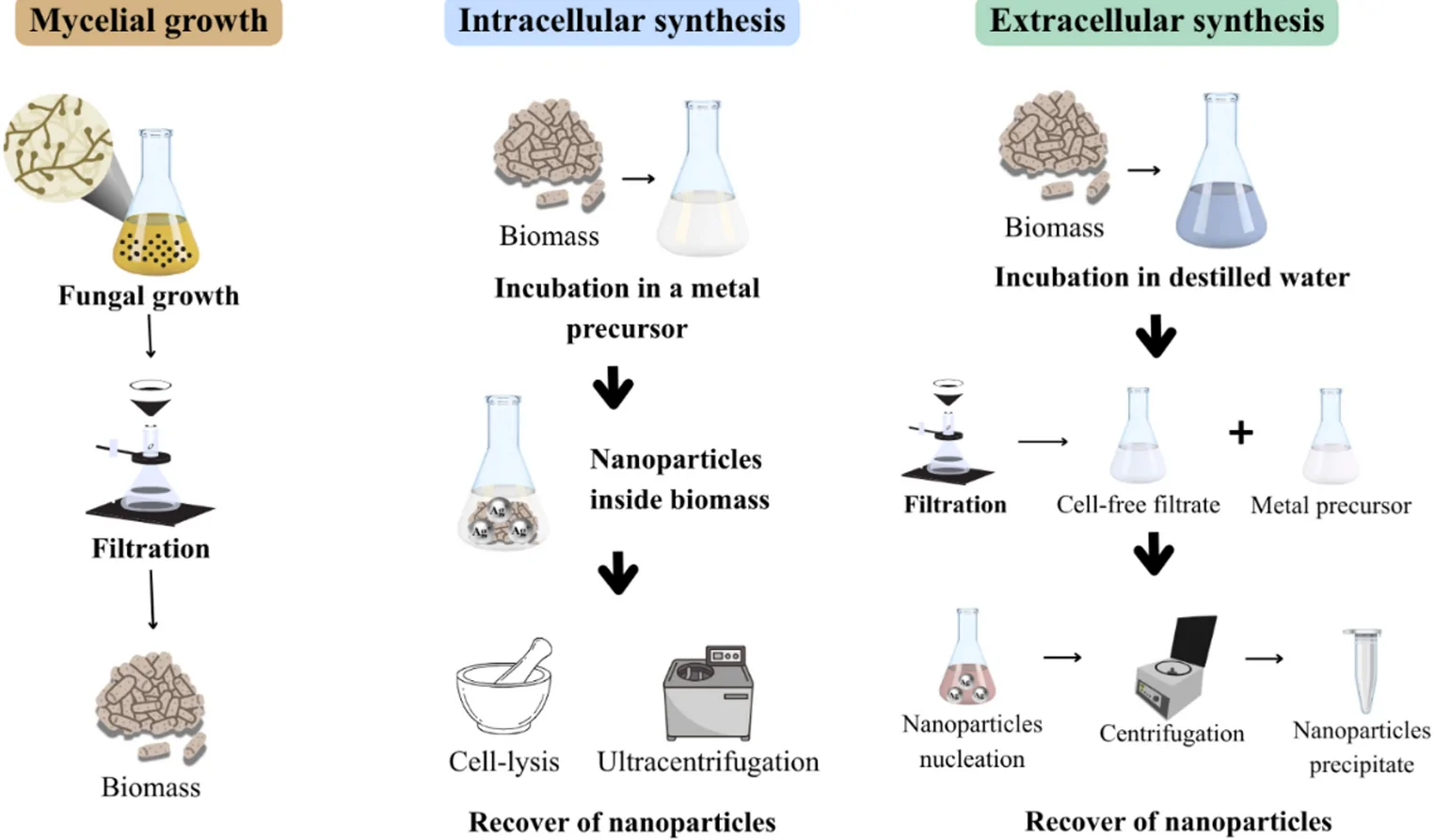
Comparison of intracellular and extracellular synthesis workflows for fungal nanoparticle synthesis

This review primarily included studies that used extracellular synthesis procedures (Fig. 6), with 3% not providing a clear description of their methodology. This method is common because it involves releasing metabolites, proteins, enzymes, peptides, and polysaccharides from the biomass through incubation, most often in distilled water. Nanoparticle formation occurs outside the cells, within the secretome, which is the mixture of secreted biomolecules in the cell-free filtrate. Essentially, metal ions are reduced and nucleated in the external medium rather than inside the cytoplasm or cell wall [27, 191].

The extracellular pathway begins when metal ions interact with a mixture of secreted biomolecules, without requiring cellular contact or internalization. Initially, metal ions are drawn to and bind with secreted proteins and polysaccharides via electrostatic interactions and surface complexation. Next, enzymes such as nitrate reductase and NADH-dependent reductase facilitate electron transfer from cofactors in the filtrate to the metal ions, thereby altering their chemical state via redox reactions [55, 192]. A crucial aspect to consider is that reduction to the elemental metal (M^0^) is not the sole pathway in nanoparticle synthesis. Fungal systems can produce nanoparticles in non-elemental forms, such as metal oxides and mixed compounds. For noble metals such as silver, which have high reduction potentials (E° = +0.80 V), enzymatic reduction yields metallic nanoparticles. Conversely, certain ions, including zinc, iron, and titanium, tend to undergo oxidation, forming metal oxides such as ZnO, FeO, and TiO2 [127, 164, 193]. To prevent aggregation and ensure colloidal stability, biomolecules secreted into the environment bind to the nanoparticle surface. These biomolecules, which include proteins, peptides, polysaccharides, and other metabolites, interact through functional groups such as amino, carboxylate, and hydroxyl groups, forming a stabilizing corona around the nanoparticles [194]. This corona acts as a natural capping layer that governs stability, dispersibility, and biological behavior of the nanoparticles [195] Extracellular synthesis occurs in the culture filtrate, enabling direct recovery of nanoparticles without disrupting the cells. Simple centrifugation or filtration of the culture supernatant is sufficient to collect nanoparticles. This process reduces operational complexity, labor, and resource consumption. Consequently, extracellular synthesis is more cost-effective and scalable than intracellular methods, making it the preferred approach for large-scale nanoparticle production [196, 197].

While the intracellular and extracellular mechanisms described above provide a structural framework for understanding fungal nanoparticle biosynthesis, the detailed molecular components, including specific genes, proteins, and metabolites involved, are still not well characterized. Addressing this knowledge gap will necessitate the application of multi-omics approaches.

### Multi-omics approaches to study fungal nanoparticle biosynthesis

**Fig. 7:**
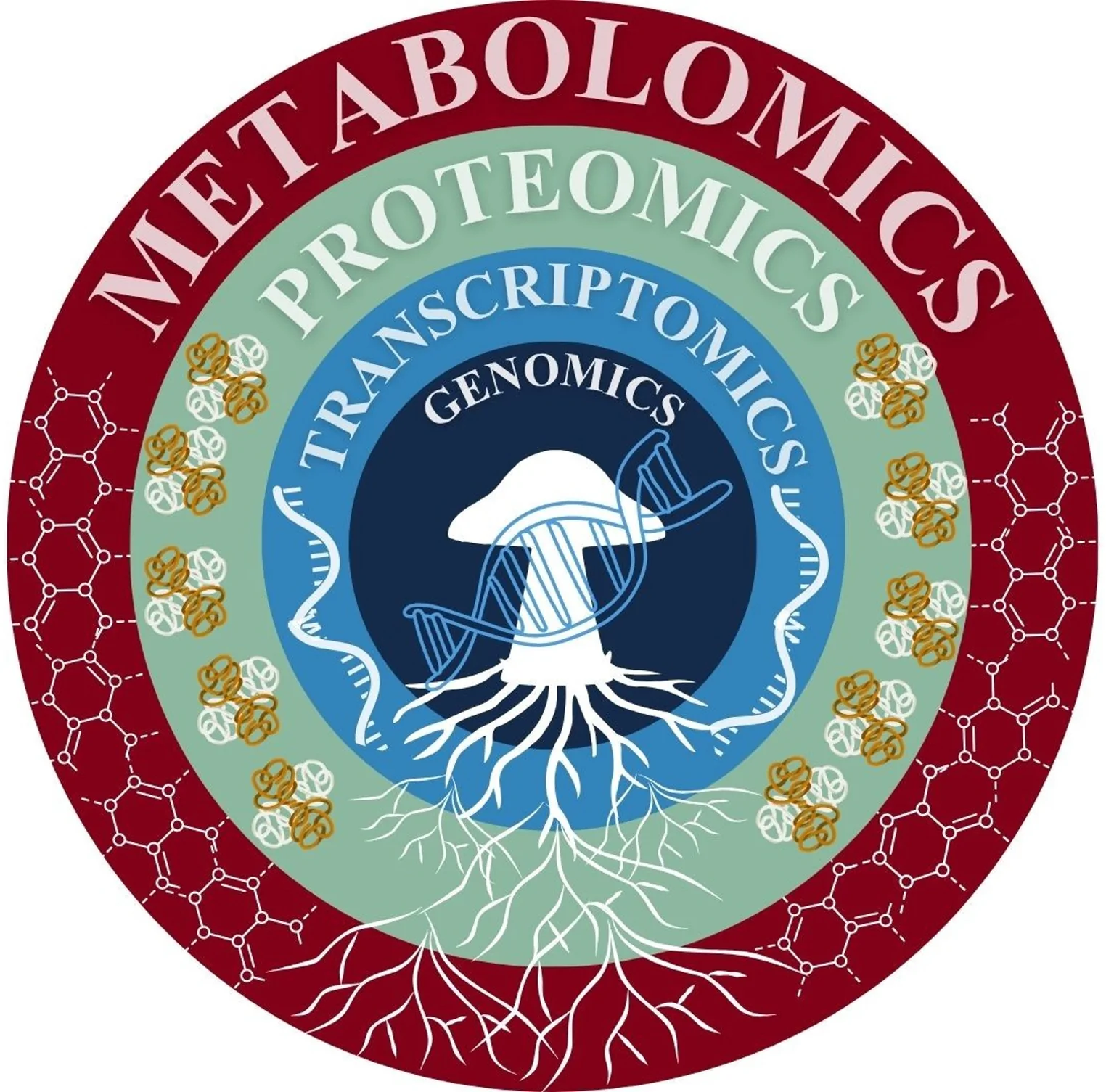
Multi-omics approaches for elucidating fungal nanoparticles synthesis

Multi-omics strategies are increasingly elucidating the molecular mechanisms underlying fungal-mediated nanoparticle synthesis (Fig. 7). The integration of metabolomics, proteomics, transcriptomics, and genomics offers insights into how fungi coordinate redox metabolism, secretion systems, and stress-responsive pathways during metal-biomolecule interactions and nanoparticle stabilization. Although the field is still in its early stages, recent studies provide a mechanistic understanding of the likely processes involved in nanoparticle formation.

Metabolomics may identify the molecules that facilitate fungal nanoparticle formation. In *Penicillium pimiteouisense*, metabolomic mapping revealed that CuO nanoparticle synthesis is supported by NADPH-dependent reductases and phenolic metabolites, as well as peptides that act as capping agents, thereby linking fungal secondary metabolism to the reduction of metal ions [198]. More broadly, metabolomic surveys across fungi can highlight stress-responsive pathways, such as trehalose cycling, gliotoxin modulation, and glyoxylate shunt activation, which often overlap with fungal metal detoxification mechanisms [199]. These metabolic signatures provide a framework for understanding how fungi produce biomolecules essential for nanoparticle nucleation. Consequently, metabolomics is a critical tool for identifying which metabolites are actively involved in synthesis and which serve as incidental byproducts.

Proteomic profiling identifies secreted proteins and enzymes that drive nanoparticle formation, particularly within the secretome. In *Trichoderma harzianum*, secretome profiling identified 99 proteins in the culture medium and 304 in the aqueous filtrate used for ZnO nanoparticle synthesis [200]. These findings constitute the first proteomic evidence linking specific proteins and enzymes in the nanoparticle synthesis, supporting the long-standing hypothesis that extracellular enzymes orchestrate fungal nanoparticle assembly.

Integrated transcriptomics and proteomics can elucidate global stress-response mechanisms that govern metal interactions. In *Penicillium* sp. exposed to selenite, an integrated database encompassing 6,175 expressed genes and 695 regulated proteins, with coordinated activation of the glutathione system, Fe-S cluster assembly machinery, lipid remodeling enzymes, and membrane transporters [201]. These responses support cellular redox balance while enabling the reduction of selenium from Se(IV) to Se^0^, a reaction fundamental to selenium nanoparticle formation. The combination of transcriptomics and proteomics approaches enables the identification of key regulatory nodes, such as transcription factors or signaling kinases, that control biosynthetic processes and are critical targets for genetic and metabolic engineering.

Genomics and bioinformatics tools have the potential to facilitate the discovery of previously unidentified biosynthetic gene clusters (BGCs). Machine learning models trained on fungal BGCs can predict the functions of secondary metabolites even in datasets with limited data [202], while recent advances in phylogenomic and long-read sequencing technologies accelerate the identification of redox-active gene families across the Ascomycota [203, 204]. Complementary bioinformatic analyses can also model the ion-binding properties of extracellular metabolites [205]. Consequently, genomics can be used to identify fungal strains with genetic potential for efficient nanoparticle biosynthesis prior to laboratory synthesis, thereby guiding strain selection. In summary, each layer of multi-omics contributes a specific function both individually and collectively. The integration of these approaches is beginning to bridge the mechanistic gap in fungal-mediated nanoparticle synthesis.

## Potential future directions

Fungal-mediated nanoparticle biosynthesis has considerable potential for sustainable nanomaterial production, but progress toward reproducible and technologically relevant processes requires a transition from descriptive studies to hypothesis-driven experimental design. Based on the methodological, taxonomic, and mechanistic gaps identified in this review, three priorities should guide future development of fungal nanotechnology.

### Establishment of causal relationship between cultivation conditions and nanoparticle properties

It is essential to identify which experimental variables directly influence nanoparticle formation and their physicochemical properties. Current research often varies multiple factors simultaneously, such as fungal strain, cultivation medium, biomass concentration, pH, temperature, incubation time, and agitation, making it challenging to attribute changes in nanoparticle yield, size, morphology, or stability to specific factors. Future investigations should adopt controlled experimental designs where these parameters are varied independently or systematically combined. Special attention should be paid to direct comparisons of cultivation media and fungal growth phases under consistent synthesis conditions. Since fungal metabolism and biomolecule secretion can vary significantly depending on strain [206], Substrate and environmental conditions make standardized reporting of experimental details essential for distinguishing biological effects from methodological variability. A persistent challenge is the lack of uniform protocols across species and laboratories, as metabolic responses and biomolecule secretion depend on strain, substrate, and environmental factors. This variability hampers reproducibility and complicates direct comparisons between studies, posing a significant barrier to technological development and industrial application. Additionally, differences in cultivation methods, such as submerged versus solid-state fermentation, can result in distinct metabolic profiles and nanoparticle characteristics.

### Validation of the specific fungal molecules responsible for nanoparticle formation

Enzymes, proteins, polysaccharides, and secondary metabolites are frequently proposed to facilitate metal reduction, nucleation, and stabilization processes. Nevertheless, their individual roles have not been conclusively demonstrated. Future research should employ integrated multi-omics approaches to move beyond correlative observations and precisely identify the fungal molecules responsible for nanoparticle formation, rather than treating fungal filtrates as uniform biosynthetic systems [207]. Specifically, combining genomics, transcriptomics, proteomics, and metabolomics with functional assays would enable differentiation between reductants and accessory capping or stabilizing agents, thereby clarifying which biomolecules influence particle size, morphology, and stability. Existing studies suggest candidate drivers such as reductases, oxidoreductases, secreted proteins, phenolic metabolites, peptides, and polysaccharides [208]; however, these findings require direct causal validation across diverse fungal species and nanoparticle types. A critical priority is to establish mechanistic links between omics-derived candidates and biosynthetic pathways, allowing the field to identify consistently responsible compounds and distinguish them from context-dependent byproducts.

### Expand fungal and metal diversity

Research efforts continue to be primarily concentrated on a limited set of fungal genera such as *Aspergillus*, *Penicillium*, and *Fusarium*, as well as on a narrow selection of metals including silver, gold, and zinc. Future screening endeavors should broaden to encompass underrepresented fungal taxa and metal oxides with potential applications in magnetic, catalytic, semiconducting, biomedical, or environmental fields. Incorporating multi-omics data could facilitate the prioritization of fungi that possess relevant redox enzymes, metal resistance pathways, extracellular secretion profiles, or metal-binding metabolites. This approach would transition the field from empirical strain screening toward biologically informed selection, potentially revealing fungal systems that are better suited to specific nanoparticle compositions or functional requirements.

Together, these priorities provide a pathway from empirical nanoparticle production toward mechanistically defined, reproducible, and scalable fungal bioprocesses. Progress in these areas would also strengthen the contribution of fungal nanotechnology to sustainable production and innovation, particularly in the context of the United Nations Sustainable Development Goals (SDGs) [209].

## Conclusions

Filamentous fungi represent a promising and sustainable platform for the biosynthesis of metallic nanoparticles, particularly silver, gold, and zinc. Genera such as *Aspergillus*, *Penicillium*, and *Fusarium* have been widely studied for their ability to produce secondary metabolites and enzymes that reduce and stabilize metal ions. Combined with their inherent adaptability, these traits position fungi as key agents in green nanotechnology. Despite this potential, significant gaps hinder reproducibility, mechanistic understanding, and industrial translation. Standardization of culture conditions, including medium composition, pH, and incubation time, remains limited, and few studies systematically address the influence of fungal morphology, growth phase, or metabolic regulation on nanoparticle yield and functionality. At the molecular level, biosynthetic mechanisms are inferred primarily from FTIR data, without integrating genomics, transcriptomics, proteomics, or metabolomics. Future research should prioritize standardized cultivation and extraction protocols, broaden searches for non-conventional fungi, apply molecular and omics tools to elucidate biosynthetic pathways, and evaluate the scaling potential and biosecurity for specific applications. Addressing these challenges will consolidate fungal nanoparticle biosynthesis as an environmentally sustainable, application-oriented alternative to conventional synthetic routes, unlocking its full potential for industrial and biomedical applications.

## Supplementary Information

Below is the link to the electronic supplementary material.

Supplementary Material 1

## Data Availability

No datasets were generated or analysed during the current study.
