# Supplementary material for: Current challenges and future directions in the green synthesis of nanoparticles using fungi
Source: Bioprocess Biosyst Eng. 2026 Sep 23;49(11):3059–84. doi: 10.1007/s00449-026-03430-7 (PMC13661679; doi:10.1007/s00449-026-03430-7)
Supplement: Supplementary file 1 [file 449_2026_3430_MOESM1_ESM.docx]

**Supplementary Materials**

Current Advances and Future Perspectives in Fungal-Mediated Nanoparticle Synthesis

**Figure S1** - Results obtained using the Preferred Reporting Items for Systematic Reviews and Meta-Analyses (PRISMA) flow diagram and the database search indicators

Table 1 – A comprehensive description of the indicators employed across three databases and the results obtained from each source.

| Database | SearchField | Number of articles found |
| --- | --- | --- |
| PubMed | ("nanoparticle" OR "nanoparticles" OR "nanotechnology") AND (nanoparticles by fungi" OR "myconanotechnology") NOT ("plant" OR "leaf" OR "bacteria" OR "yeast" OR "treatment of fungi" OR "pathogenicity") | 327 |
| Scopus | ("nanoparticle" OR "nanoparticles" OR "nanotechnology") AND (nanoparticles by fungi" OR "myconanotechnology") NOT ("plant" OR "leaf" OR "bacteria" OR "yeast" OR "treatment of fungi" OR "pathogenicity") | 494 |
| Web of Science | ("nanoparticle" OR "nanoparticles" OR "nanotechnology") AND (nanoparticles by fungi" OR "myconanotechnology") NOT ("plant" OR "leaf" OR "bacteria" OR "yeast" OR "treatment of fungi" OR "pathogenicity") | 41 |

**Table S2** - Supplementary References of Studies Eligible for the Review

Abd El-Aziz, A.R.M., Al-Othman, M.R., Mahmoud, M.A. and Metwaly, H.A., 2015. Biosynthesis of silver nanoparticles using Fusarium solani and its impact on grain borne fungi. Digest Journal of Nanomaterials and Biostructures, .

Abd Elghaffar, R.Y., Emam, A.M., Taher, E.S., Baz, M.M., Nayel, H., Abdeen, A., El-Nablaway, M., Alwutayd, K.M., Mihaela, O., Ioan, B.-D., Khattab, A.A., Al-Serwi, R.H. and Sehim, A.E., 2024. The potential biological activities of Aspergillus luchuensis-aided green synthesis of silver nanoparticles. Frontiers in microbiology, 15, p.1381302. https://doi.org/10.3389/fmicb.2024.1381302.

Abdelkader, D.H., Negm, W.A., Elekhnawy, E., Eliwa, D., Aldosari, B.N. and Almurshedi, A.S., 2022. Zinc Oxide Nanoparticles as Potential Delivery Carrier: Green Synthesis by Aspergillus niger Endophytic Fungus, Characterization, and In Vitro/In Vivo Antibacterial Activity. Pharmaceuticals (Basel, Switzerland), 15(9). https://doi.org/10.3390/ph15091057.

Abdelrahman, S.E.S.A.H., El Hawary, S., Mohsen, E., El Raey, M.A., Selim, H.M.R.M., Hamdan, A.M.E., Ghareeb, M.A. and Hamed, A.A., 2024. Bio-fabricated zinc oxide nanoparticles mediated by endophytic fungus Aspergillus sp. SA17 with antimicrobial and anticancer activities: in vitro supported by in silico studies. Frontiers in microbiology, 15, p.1366614. https://doi.org/10.3389/fmicb.2024.1366614.

Abdullah, N.H., 2022. Optimization of magnetic nano-iron production by Aspergillus flavipes MN956655.1 using response surface methodology and evaluation of their dye decolorizing and antifungal activities. Scientific reports, 12(1), p.21059. https://doi.org/10.1038/s41598-022-25339-3.

Abu-Tahon, M.A., Alshammari, F.A., Shahhat, I.M., Ghareib, M. and Abdallah, W.E., 2025. Eco-Friendly Synthesis, Characterization, and Biomedical Applications of Biosynthesized Bimetallic Silver-Gold Nanoparticles by Culture Supernatant of Aspergillus niger. Applied biochemistry and biotechnology, 197(1), pp.137–158. https://doi.org/10.1007/s12010-024-05035-w.

Ahmed, A.-A., Hamzah, H. and Maaroof, M., 2018. Analyzing formation of silver nanoparticles from the filamentous fungus Fusarium oxysporum and their antimicrobial activity. TURKISH JOURNAL OF BIOLOGY, 42, pp.54–62. https://doi.org/10.3906/biy-1710-2.

Akther, T. and Hemalatha, S., 2019. Mycosilver Nanoparticles: Synthesis, Characterization and its Efficacy against Plant Pathogenic Fungi. BioNanoScience, https://doi.org/10.1007/s12668-019-0607-y.

Albalawi, M.A., Abdelaziz, A.M., Attia, M.S., Saied, E., Elganzory, H.H. and Hashem, A.H., 2022. Mycosynthesis of Silica Nanoparticles Using Aspergillus niger: Control of Alternaria solani Causing Early Blight Disease, Induction of Innate Immunity and Reducing of Oxidative Stress in Eggplant. Antioxidants (Basel, Switzerland), 11(12). https://doi.org/10.3390/antiox11122323.

Al-Enazi, N.M., Alsamhary, K. and Ameen, F., 2024. Optimized synthesis of silver nanoparticles using the marine fungus Aspergillus terreus and its application against resistant nosocomial pathogens. Biotechnology & genetic engineering reviews, 40(2), pp.1082–1093. https://doi.org/10.1080/02648725.2023.2191381.

Ali, E.M., Rajendran, P. and Abdallah, B.M., 2024. Mycosynthesis of silver nanoparticles from endophytic Aspergillus parasiticus and their antibacterial activity against methicillin-resistant Staphylococcus aureus in vitro and in vivo. Frontiers in microbiology, 15, p.1483637. https://doi.org/10.3389/fmicb.2024.1483637.

Almeida, É.S., de Oliveira, D. and Hotza, D., 2017. Characterization of silver nanoparticles produced by biosynthesis mediated by Fusarium oxysporum under different processing conditions. Bioprocess and biosystems engineering, 40(9), pp.1291–1303. https://doi.org/10.1007/s00449-017-1788-9.

Alqaraleh, M., Khleifat, K.M., Abu Hajleh, M.N., Farah, H.S. and Ahmed, K.A.-A., 2023. Fungal-Mediated Silver Nanoparticle and Biochar Synergy against Colorectal Cancer Cells and Pathogenic Bacteria. Antibiotics (Basel, Switzerland), 12(3). https://doi.org/10.3390/antibiotics12030597.

Alves, M.F., Paschoal, A.C.C., Klimeck, T.D.F., Kuligovski, C., Marcon, B.H., de Aguiar, A.M. and Murray, P.G., 2022. Biological Synthesis of Low Cytotoxicity Silver Nanoparticles (AgNPs) by the Fungus Chaetomium thermophilum— Sustainable Nanotechnology. Journal of Fungi, https://doi.org/10.3390/jof8060605.

Ameen, F., 2022. Optimization of the Synthesis of Fungus‐Mediated Bi‐Metallic Ag‐Cu Nanoparticles. Applied Sciences (Switzerland), https://doi.org/10.3390/app12031384.

Ameen, F., Al-Homaidan, A.A., Al-Sabri, A., Almansob, A. and AlNAdhari, S., 2023. Anti-oxidant, anti-fungal and cytotoxic effects of silver nanoparticles synthesized using marine fungus Cladosporium halotolerans. Applied Nanoscience (Switzerland), https://doi.org/10.1007/s13204-021-01874-9.

Ammar, H.A., Alghazaly, M.S., Assem, Y. and Abou Zeid, A.A., 2021. Bioengineering and optimization of PEGylated zinc nanoparticles by simple green method using Monascus purpureus, and their powerful antifungal activity against the most famous plant pathogenic fungi, causing food spoilage. Environmental Nanotechnology, Monitoring and Management, https://doi.org/10.1016/j.enmm.2021.100543.

Ammar, H.A., Rabie, G.H. and Mohamed, E., 2019. Novel fabrication of gelatin-encapsulated copper nanoparticles using Aspergillus versicolor and their application in controlling of rotting plant pathogens. Bioprocess and biosystems engineering, 42(12), pp.1947–1961. https://doi.org/10.1007/s00449-019-02188-5.

Ammar, H.A.M. and El-Desouky, T.A., 2016. Green synthesis of nanosilver particles by Aspergillus terreus HA1N and Penicillium expansum HA2N and its antifungal activity against mycotoxigenic fungi. Journal of applied microbiology, 121(1), pp.89–100. https://doi.org/10.1111/jam.13140.

Andrade, F., Jenipher, C., Gurav, N., Nadaf, S., Khan, M.S., Kalaskar, M., Bhinge, S., Bhole, R., Ayyanar, M. and Gurav, S., 2024a. Endophytic Fungus Colletotrichum siamense Derived Silver Nanoparticles: Biomimetic Synthesis, Process Optimization and Their Biomedical Applications. Journal of Inorganic and Organometallic Polymers and Materials. https://doi.org/10.1007/s10904-024-03235-9.

Andrade, F., Jenipher, C., Gurav, N., Nadaf, S., Khan, M.S., Mahajan, N., Bhagwat, D., Kalaskar, M., Chikhale, R., Bhole, R., Lalsare, S., Baheti, A., Ayyanar, M. and Gurav, S., 2024b. Endophytic fungi-assisted biomass synthesis of eco-friendly formulated silver nanoparticles for enhanced antibacterial, antioxidant, and antidiabetic activities. Journal of Drug Delivery Science and Technology, https://doi.org/10.1016/j.jddst.2024.105749.

Ao, B., He, F., Lv, J., Tu, J., Tan, Z., Jiang, H., Shi, X., Li, J., Hou, J., Hu, Y. and Xia, X., 2022. Green synthesis of biogenetic Te(0) nanoparticles by high tellurite tolerance fungus Mortierella sp. AB1 with antibacterial activity. Frontiers in Microbiology, https://doi.org/10.3389/fmicb.2022.1020179.

Awad, M.A., Eid, A.M., Elsheikh, T.M.Y., Al-Faifi, Z.E., Saad, N., Sultan, M.H., Selim, S., Al-Khalaf, A.A. and Fouda, A., 2022. Mycosynthesis, Characterization, and Mosquitocidal Activity of Silver Nanoparticles Fabricated by Aspergillus niger Strain. Journal of fungi (Basel, Switzerland), 8(4). https://doi.org/10.3390/jof8040396.

Balakumaran, M.D., Ramachandran, R. and Kalaichelvan, P.T., 2015. Exploitation of endophytic fungus, Guignardia mangiferae for extracellular synthesis of silver nanoparticles and their in vitro biological activities. Microbiological Research, https://doi.org/10.1016/j.micres.2015.05.009.

Barabadi, H., Kobarfard, F. and Vahidi, H., 2018. Biosynthesis and Characterization of Biogenic Tellurium Nanoparticles by Using Penicillium chrysogenum PTCC 5031: A Novel Approach in Gold Biotechnology. Iranian journal of pharmaceutical research : IJPR, 17(Suppl2), pp.87–97.

Barabadi, H., Mobaraki, K., Jounaki, K., Sadeghian-Abadi, S., Vahidi, H., Jahani, R., Noqani, H., Hosseini, O., Ashouri, F. and Amidi, S., 2023. Exploring the biological application of Penicillium fimorum-derived silver nanoparticles: In vitro physicochemical, antifungal, biofilm inhibitory, antioxidant, anticoagulant, and thrombolytic performance. Heliyon, 9(6), p.e16853. https://doi.org/10.1016/j.heliyon.2023.e16853.

Barapatre, A., Aadil, K.R. and Jha, H., 2016. Synergistic antibacterial and antibiofilm activity of silver nanoparticles biosynthesized by lignin-degrading fungus. Bioresources and Bioprocessing, https://doi.org/10.1186/s40643-016-0083-y.

Barbosa, A.C.M.S., Costa Silva, L.P., Ferraz, C.M., Tobias, F.L., De Araújo, J.V., Loureiro, B., Braga, G.M.A.M., Veloso, F.B.R., Soares, F.E.D.F., Fronza, M. and Braga, F.R., 2019. Nematicidal activity of silver nanoparticles from the fungus Duddingtonia flagrans. International Journal of Nanomedicine, https://doi.org/10.2147/IJN.S193679.

Basheer, M.A., Abutaleb, K., Abed, N.N. and Mekawey, A.A.I., 2023. Mycosynthesis of silver nanoparticles using marine fungi and their antimicrobial activity against pathogenic microorganisms. Journal of Genetic Engineering and Biotechnology, https://doi.org/10.1186/s43141-023-00572-z.

Basu, A., Ray, S., Chowdhury, S., Sarkar, A., Mandal, D.P., Bhattacharjee, S. and Kundu, S., 2018. Evaluating the antimicrobial, apoptotic, and cancer cell gene delivery properties of protein-capped gold nanoparticles synthesized from the edible mycorrhizal fungus Tricholoma crassum. Nanoscale Research Letters, https://doi.org/10.1186/s11671-018-2561-y.

Bedi, A., Singh, B.R., Deshmukh, S.K., Adholeya, A. and Barrow, C.J., 2018. An Aspergillus aculateus strain was capable of producing agriculturally useful nanoparticles via bioremediation of iron ore tailings. Journal of Environmental Management, 215, pp.100–107. https://doi.org/10.1016/j.jenvman.2018.03.049.

Bhandari, Y., Varma, S., Sawant, A., Beemagani, S., Jaiswal, N., Chaudhari, B.P. and Vamkudoth, K.R., 2023. Biosynthesis of gold nanoparticles by Penicillium rubens and catalytic detoxification of ochratoxin A and organic dye pollutants. International microbiology : the official journal of the Spanish Society for Microbiology, 26(4), pp.765–780. https://doi.org/10.1007/s10123-023-00341-5.

Bhargava, A., Jain, N., Khan, M.A., Pareek, V., Dilip, R.V. and Panwar, J., 2016. Utilizing metal tolerance potential of soil fungus for efficient synthesis of gold nanoparticles with superior catalytic activity for degradation of rhodamine B. Journal of Environmental Management, https://doi.org/10.1016/j.jenvman.2016.08.021.

Bhattacharjee, S., Debnath, G., Das, A.R., Saha, A.K. and Das, P., 2017. Characterization of silver nanoparticles synthesized using an endophytic fungus, Penicillium oxalicum having potential antimicrobial activity. Advances in Natural Sciences: Nanoscience and Nanotechnology, https://doi.org/10.1088/2043-6254/aa84ec.

Boldt, A., Walter, J., Hofbauer, F., Stetter, K., Aubel, I., Bertau, M., Jäger, C.M. and Walther, T., 2023. Cell-free synthesis of silver nanoparticles in spent media of different Aspergillus species. Engineering in life sciences, 23(3), p.e202200052. https://doi.org/10.1002/elsc.202200052.

Chandankere, R., Chelliah, J., Subban, K., Shanadrahalli, V.C., Parvez, A., Zabed, H.M., Sharma, Y.C. and Qi, X., 2020. Pleiotropic Functions and Biological Potentials of Silver Nanoparticles Synthesized by an Endophytic Fungus. Frontiers in Bioengineering and Biotechnology, https://doi.org/10.3389/fbioe.2020.00095.

Chatterjee, S., Mahanty, S., Das, P., Chaudhuri, P. and Das, S., 2020. Biofabrication of iron oxide nanoparticles using manglicolous fungus Aspergillus niger BSC-1 and removal of Cr(VI) from aqueous solution. Chemical Engineering Journal, https://doi.org/10.1016/j.cej.2019.123790.

Chen, H., Luo, L., Fan, S., Xiong, Y., Ling, Y. and Peng, S., 2021. Zinc oxide nanoparticles synthesized from Aspergillus terreus induces oxidative stress-mediated apoptosis through modulating apoptotic proteins in human cervical cancer HeLa cells. The Journal of pharmacy and pharmacology, 73(2), pp.221–232. https://doi.org/10.1093/jpp/rgaa043.

Clarance, P., Luvankar, B., Sales, J., Khusro, A., Agastian, P., Tack, J.-C., Al Khulaifi, M.M., Al-Shwaiman, H.A., Elgorban, A.M., Syed, A. and Kim, H.-J., 2020. Green synthesis and characterization of gold nanoparticles using endophytic fungi Fusarium solani and its in-vitro anticancer and biomedical applications. Saudi journal of biological sciences, 27(2), pp.706–712. https://doi.org/10.1016/j.sjbs.2019.12.026.

Costa Silva, L.P., Pinto Oliveira, J., Keijok, W.J., da Silva, A.R., Aguiar, A.R., Guimarães, M.C.C., Ferraz, C.M., Araújo, J.V., Tobias, F.L. and Braga, F.R., 2017. Extracellular biosynthesis of silver nanoparticles using the cell-free filtrate of nematophagous fungus Duddingtonia flagrans. International Journal of Nanomedicine, https://doi.org/10.2147/IJN.S137703.

Dadayya, M., Gowri Thippeswamy, M., Shivaiah, N., Hirakannavar Veeranna, S., Gurubasajar, N., Subhakar, A. and Basaiah, T., 2023. Biological Potential of Silver Nanoparticles Synthesized by an Endophytic Fungus Metapochonia suchlasporia-KUMBMDBT-23. BioNanoScience, https://doi.org/10.1007/s12668-023-01177-z.

Dias, C., Ayyanar, M., Amalraj, S., Khanal, P., Subramaniyan, V., Das, S., Gandhale, P., Biswa, V., Ali, R., Gurav, N., Nadaf, S., Rarokar, N. and Gurav, S., 2022. Biogenic synthesis of zinc oxide nanoparticles using mushroom fungus Cordyceps militaris: Characterization and mechanistic insights of therapeutic investigation. Journal of Drug Delivery Science and Technology, 73, p.103444. https://doi.org/10.1016/j.jddst.2022.103444.

El Dougdoug, N.K., Attia, M.S., Malash, M.N., Abdel-Maksoud, M.A., Malik, A., Kiani, B.H., Fesal, A.A., Rizk, S.H., El-Sayyad, G.S. and Harb, N., 2025. Aspergillus fumigatus-induced biogenic silver nanoparticles’ efficacy as antimicrobial and antibiofilm agents with potential anticancer activity: An in vitro investigation. Microbial Pathogenesis, 199, p.106950. https://doi.org/10.1016/j.micpath.2024.106950.

El Sayed, M.T. and El-Sayed, A.S.A., 2020. Biocidal Activity of Metal Nanoparticles Synthesized by Fusarium solani against Multidrug-Resistant Bacteria and Mycotoxigenic Fungi. Journal of microbiology and biotechnology, 30(2), pp.226–236. https://doi.org/10.4014/jmb.1906.06070.

Elamawi, R.M., Al-Harbi, R.E. and Hendi, A.A., 2018. Biosynthesis and characterization of silver nanoparticles using Trichoderma longibrachiatum and their effect on phytopathogenic fungi. Egyptian Journal of Biological Pest Control, https://doi.org/10.1186/s41938-018-0028-1.

Elangovan, U., Anbarasu, K., Shanmugam, R., Veeramuthu, D. and John, J.G., 2024. Biological Properties of Mycosynthesized Novel Strontium Oxide Nanoparticles from Biomass Free Filtrate of Endophytic Fungus Aspergillus sp. LCJ315. BioNanoScience, https://doi.org/10.1007/s12668-024-01499-6.

El-Sharkawy, R.M. and Abbas, M.H.H., 2023. Unveiling antibacterial and antioxidant activities of zinc phosphate-based nanosheets synthesized by Aspergillus fumigatus and its application in sustainable decolorization of textile wastewater. BMC microbiology, 23(1), p.358. https://doi.org/10.1186/s12866-023-03054-x.

El-Sharkawy, R.M., Khairy, M., Zaki, M.E.A. and Al-Tabbakh, A.-S.M., 2025. Endophytic Aspergillus japonicus mediated biosynthesises of magnesium oxide nanoparticles: sustainable dye removal and in silico molecular docking evaluation of their enhanced antibacterial activity. Microbial cell factories, 24(1), p.44. https://doi.org/10.1186/s12934-025-02648-6.

Es-haghi, A., Taghavizadeh Yazdi, M.E., Sharifalhoseini, M., Baghani, M., Yousefi, E., Rahdar, A. and Baino, F., 2021. Application of Response Surface Methodology for Optimizing the Therapeutic Activity of ZnO Nanoparticles Biosynthesized from Aspergillus niger. Biomimetics, 6(2), p.34. https://doi.org/10.3390/biomimetics6020034.

Fatima, F., Bajpai, P., Pathak, N., Singh, S., Priya, S. and Verma, S.R., 2015. Antimicrobial and immunomodulatory efficacy of extracellularly synthesized silver and gold nanoparticles by a novel phosphate solubilizing fungus Bipolaris tetramera. BMC Microbiology, https://doi.org/10.1186/s12866-015-0391-y.

Gemishev, O.T., Panayotova, M.I., Mintcheva, N.N., Djerahov, L.P., Tyuliev, G.T. and Gicheva, G.D., 2019. A green approach for silver nanoparticles preparation by cell-free extract from Trichoderma reesei fungi and their characterization. Materials Research Express, 6(9), p.095040. https://doi.org/10.1088/2053-1591/ab2e6a.

Gharieb, M.M., Soliman, Azza.M. and Omara, M.S., 2025. Biosynthesis of selenium nanoparticles by potential endophytic fungi Penicillium citrinum and Rhizopus arrhizus: characterization and maximization. Biomass Conversion and Biorefinery, https://doi.org/10.1007/s13399-023-05084-x.

Gudikandula, K., Vadapally, P. and Singara Charya, M.A., 2017. Biogenic synthesis of silver nanoparticles from white rot fungi: Their characterization and antibacterial studies. OpenNano, 2, pp.64–78. https://doi.org/10.1016/j.onano.2017.07.002.

Gupta, K. and Chundawat, T.S., 2019. Bio-inspired synthesis of platinum nanoparticles from fungus Fusarium oxysporum : its characteristics, potential antimicrobial, antioxidant and photocatalytic activities. Materials Research Express, 6(10), p.1050d6. https://doi.org/10.1088/2053-1591/ab4219.

Hamad, M.T., 2018. Biosynthesis of silver nanoparticles by fungi and their antibacterial activity. International Journal of Environmental Science and Technology, https://doi.org/10.1007/s13762-018-1814-8.

Hashem, A.H., Khalil, A.M.A., Reyad, A.M. and Salem, S.S., 2021. Biomedical Applications of Mycosynthesized Selenium Nanoparticles Using Penicillium expansum ATTC 36200. Biological trace element research, 199(10), pp.3998–4008. https://doi.org/10.1007/s12011-020-02506-z.

Ilahi, N., Haleem, A., Iqbal, S., Fatima, N., Sajjad, W., Sideeq, A. and Ahmed, S., 2022. Biosynthesis of silver nanoparticles using endophytic Fusarium oxysporum strain NFW16 and their in vitro antibacterial potential. Microscopy research and technique, 85(4), pp.1568–1579. https://doi.org/10.1002/jemt.24018.

Janakiraman, V., Govindarajan, K. and Magesh, C.R., 2019. Biosynthesis of Silver Nanoparticles from Endophytic Fungi, and its Cytotoxic Activity. BioNanoScience, https://doi.org/10.1007/s12668-019-00631-1.

Kathiravan, A., Udayan, E., Rajeshkumar, S. and Gnanadoss, J.J., 2023. Unveiling the Biological Potential of Mycosynthesized Selenium Nanoparticles from Endophytic Fungus Curvularia sp. LCJ413. BioNanoScience, https://doi.org/10.1007/s12668-023-01223-w.

Khalil, N.M., Abd El-Ghany, M.N. and Rodríguez-Couto, S., 2019. Antifungal and anti-mycotoxin efficacy of biogenic silver nanoparticles produced by Fusarium chlamydosporum and Penicillium chrysogenum at non-cytotoxic doses. Chemosphere, 218, pp.477–486. https://doi.org/10.1016/j.chemosphere.2018.11.129.

Li, Q. and Gadd, G.M., 2017. Biosynthesis of copper carbonate nanoparticles by ureolytic fungi. Applied Microbiology and Biotechnology, https://doi.org/10.1007/s00253-017-8451-x.

Li, Z., Wu, F. and Zhang, Q., 2025. Zinc oxide nanoparticles derived from Penicillium griseofulvum mitigate DMBA/TPA-promoted mice skin carcinogenesis by modulating NF-ĸB associated signalling. Naunyn-Schmiedeberg’s archives of pharmacology, 398(1), pp.891–902. https://doi.org/10.1007/s00210-024-03311-4.

Liu, F., Csetenyi, L. and Gadd, G.M., 2019. Amino acid secretion influences the size and composition of copper carbonate nanoparticles synthesized by ureolytic fungi. Applied Microbiology and Biotechnology, https://doi.org/10.1007/s00253-019-09961-2.

Lotfy, W.A., Alkersh, B.M., Sabry, S.A. and Ghozlan, H.A., 2021. Biosynthesis of Silver Nanoparticles by Aspergillus terreus: Characterization, Optimization, and Biological Activities. Frontiers in bioengineering and biotechnology, 9, p.633468. https://doi.org/10.3389/fbioe.2021.633468.

M, M.H., Joshi, C.G., Danagoudar, A., Poyya, J., Kudva, A.K. and Bl, D., 2017. Biogenic synthesis of gold nanoparticles by marine endophytic fungus-Cladosporium cladosporioides isolated from seaweed and evaluation of their antioxidant and antimicrobial properties. Process Biochemistry, 63, pp.137–144. https://doi.org/10.1016/j.procbio.2017.09.008.

Majeed, S., Abdullah, M.S.B., Dash, G.K., Ansari, M.T. and Nanda, A., 2016. Biochemical synthesis of silver nanoprticles using filamentous fungi Penicillium decumbens (MTCC-2494) and its efficacy against A-549 lung cancer cell line. Chinese journal of natural medicines, 14(8), pp.615–620. https://doi.org/10.1016/S1875-5364(16)30072-3.

Manjunath, H.M., Joshi, C.G. and Raju, N.G., 2017. Biofabrication of gold nanoparticles using marine endophytic fungus - Penicillium citrinum. IET Nanobiotechnol, 11(1), pp.40–44. https://doi.org/10.1049/iet-nbt.2016.0065.

Mishra, R.C., Kalra, R., Dilawari, R., Goel, M. and Barrow, C.J., 2022. Bio-Synthesis of Aspergillus terreus Mediated Gold Nanoparticle: Antimicrobial, Antioxidant, Antifungal and In Vitro Cytotoxicity Studies. Materials (Basel, Switzerland), 15(11). https://doi.org/10.3390/ma15113877.

Mistry, H., Thakor, R. and Bariya, H., 2022. Biogenesis and characterization of proficient silver nanoparticles employing marine procured fungi Hamigera pallida and assessment of their antioxidative, antimicrobial and anticancer potency. Biotechnology Letters, https://doi.org/10.1007/s10529-022-03287-2.

Mistry, H., Thakor, R., Patil, C., Trivedi, J. and Bariya, H., 2021. Biogenically proficient synthesis and characterization of silver nanoparticles employing marine procured fungi Aspergillus brunneoviolaceus along with their antibacterial and antioxidative potency. Biotechnology letters, 43(1), pp.307–316. https://doi.org/10.1007/s10529-020-03008-7.

Mohammed, E.J., Abdelaziz, A.E.M., Mekky, A.E., Mahmoud, N.N., Sharaf, M., Al-Habibi, M.M., Khairy, N.M., Al-Askar, A.A., Youssef, F.S., Gaber, M.A., Saied, E., AbdElgayed, G., Metwally, S.A. and Shoun, A.A., 2024. Biomedical Promise of Aspergillus Flavus-Biosynthesized Selenium Nanoparticles: A Green Synthesis Approach to Antiviral, Anticancer, Anti-Biofilm, and Antibacterial Applications. Pharmaceuticals (Basel, Switzerland), 17(7). https://doi.org/10.3390/ph17070915.

Nanda, A., Nayak, B.K. and Krishnamoorthy, M., 2018. Antimicrobial properties of biogenic silver nanoparticles synthesized from phylloplane fungus, Aspergillus tamarii. Biocatalysis and Agricultural Biotechnology, https://doi.org/10.1016/j.bcab.2018.08.002.

Nayak, B.K., Nanda, A. and Prabhakar, V., 2018. Biogenic synthesis of silver nanoparticle from wasp nest soil fungus, Penicillium italicum and its analysis against multi drug resistance pathogens. Biocatalysis and Agricultural Biotechnology, 16, pp.412–418. https://doi.org/10.1016/j.bcab.2018.09.014.

Netala, V., Bethu, M., Pushpalatha, B., Baki, V., Aishwarya, S., Rao, J. and Tartte, V., 2016. Biogenesis of silver nanoparticles using endophytic fungus Pestalotiopsis microspora and evaluation of their antioxidant and anticancer activities. International Journal of Nanomedicine, Volume 11, pp.5683–5696. https://doi.org/10.2147/IJN.S112857.

Olvera-Aripez, J., Camacho-López, S., Flores-Castañeda, M., Belman-Rodríguez, C., Vilchis-Nestor, A.R. and Castro-Longoria, E., 2024. Biosynthesis of gold nanoparticles by fungi and its potential in SERS. Bioprocess and Biosystems Engineering, https://doi.org/10.1007/s00449-024-03053-w.

Ottoni, C.A., Maria, D.A., Gonçalves, P.J.R. de O., de Araújo, W.L. and de Souza, A.O., 2019. Biogenic Aspergillus tubingensis silver nanoparticles’ in vitro effects on human umbilical vein endothelial cells, normal human fibroblasts, HEPG2, and Galleria mellonella. Toxicology research, 8(6), pp.789–801. https://doi.org/10.1039/c9tx00091g.

Pareek, V., Bhargava, A. and Panwar, J., 2020. Biomimetic approach for multifarious synthesis of nanoparticles using metal tolerant fungi: A mechanistic perspective. Materials Science and Engineering: B, https://doi.org/10.1016/j.mseb.2020.114771.

Qaralleh, H., Khleifat, K.M., Al-Limoun, M.O., Alzedaneen, F.Y. and Al-Tawarah, N., 2019. Antibacterial and synergistic effect of biosynthesized silver nanoparticles using the fungi Tritirachium oryzae W5H with essential oil of Centaurea damascena to enhance conventional antibiotics activity. Advances in Natural Sciences: Nanoscience and Nanotechnology, 10(2), p.025016. https://doi.org/10.1088/2043-6254/ab2867.

Rodríguez-Serrano, C., Guzmán-Moreno, J., Ángeles-Chávez, C., Rodríguez-González, V., Ortega-Sigala, J.J., Ramírez-Santoyo, R.M. and Vidales-Rodríguez, L.E., 2020. Biosynthesis of silver nanoparticles by Fusarium scirpi and its potential as antimicrobial agent against uropathogenic Escherichia coli biofilms. PloS one, 15(3), p.e0230275. https://doi.org/10.1371/journal.pone.0230275.

Sarsar, V., Selwal, M.K. and Selwal, K.K., 2016. Biogenic synthesis, optimisation and antibacterial efficacy of extracellular silver nanoparticles using novel fungal isolate Aspergillus fumigatus MA. IET nanobiotechnology, 10(4), pp.215–221. https://doi.org/10.1049/iet-nbt.2015.0058.

Saxena, J., Sharma, P. and Singh, A., 2017. Biomimetic synthesis of AgNPs from Penicillium chrysogenum strain FGCC/BLS1 by optimising physico-cultural conditions and assessment of their antimicrobial potential. IET nanobiotechnology, 11(5), pp.576–583. https://doi.org/10.1049/iet-nbt.2016.0097.

Seetharaman, P.K., Chandrasekaran, R., Gnanasekar, S., Chandrakasan, G., Gupta, M., Manikandan, D.B. and Sivaperumal, S., 2018. Antimicrobial and larvicidal activity of eco-friendly silver nanoparticles synthesized from endophytic fungi Phomopsis liquidambaris. Biocatalysis and Agricultural Biotechnology, https://doi.org/10.1016/j.bcab.2018.07.006.

Sharma, J.L., Dhayal, V. and Sharma, R.K., 2021. White-rot fungus mediated green synthesis of zinc oxide nanoparticles and their impregnation on cellulose to develop environmental friendly antimicrobial fibers. 3 Biotech, https://doi.org/10.1007/s13205-021-02840-6.

Syed, A., Al Saedi, M.H., Bahkali, A.H., Elgorban, A.M., Kharat, M., Pai, K., Ghodake, G. and Ahmad, A., 2021. Biological synthesis of α-Ag2S composite nanoparticles using the fungus Humicola sp. and its biomedical applications. Journal of Drug Delivery Science and Technology, https://doi.org/10.1016/j.jddst.2021.102770.

Thakor, R., Mistry, H., Patel, H., Jhala, D., Parmar, N. and Bariya, H., 2022. Biogenic synthesis of silver nanoparticles mediated by the consortium comprising the marine fungal filtrates of Penicillium oxalicum and Fusarium hainanense along with their antimicrobial, antioxidant, larvicidal and anticancer potency. Journal of Applied Microbiology, 133(2), pp.857–869. https://doi.org/10.1111/jam.15611.

Trzcińska-Wencel, J., Wypij, M., Terzyk, A.P., Rai, M. and Golińska, P., 2023. Biofabrication of novel silver and zinc oxide nanoparticles from Fusarium solani IOR 825 and their potential application in agriculture as biocontrol agents of phytopathogens, and seed germination and seedling growth promoters. Frontiers in chemistry, 11, p.1235437. https://doi.org/10.3389/fchem.2023.1235437.

Uddandarao, P., Balakrishnan, R.M., Ashok, A., Swarup, S. and Sinha, P., 2019. Bioinspired ZnS:Gd Nanoparticles Synthesized from an Endophytic Fungi Aspergillus flavus for Fluorescence-Based Metal Detection. Biomimetics (Basel, Switzerland), 4(1). https://doi.org/10.3390/biomimetics4010011.

Vala, A.K., 2015. Exploration on green synthesis of gold nanoparticles by a marine-derived fungus Aspergillus sydowii. Environmental Progress and Sustainable Energy, https://doi.org/10.1002/ep.11949.

Vellingiri, M.M., Ashwin, J.K.M., Soundari, A.J.P.G., Sathiskumar, S., Priyadharshini, U., Paramasivam, D., Liu, W.-C. and Balasubramanian, B., 2021. Mycofabrication of AgONPs derived from Aspergillus terreus FC36AY1 and its potent antimicrobial, antioxidant, and anti-angiogenesis activities. Molecular biology reports, 48(12), pp.7933–7946. https://doi.org/10.1007/s11033-021-06824-w.

Vetchinkina, E., Loshchinina, E., Kupryashina, M., Burov, A. and Nikitina, V., 2019. Shape and Size Diversity of Gold, Silver, Selenium, and Silica Nanoparticles Prepared by Green Synthesis Using Fungi and Bacteria. Industrial and Engineering Chemistry Research, https://doi.org/10.1021/acs.iecr.9b03345.

Wang, D., Xue, B., Wang, L., Zhang, Y., Liu, L. and Zhou, Y., 2021. Fungus-mediated green synthesis of nano-silver using Aspergillus sydowii and its antifungal/antiproliferative activities. Scientific reports, 11(1), p.10356. https://doi.org/10.1038/s41598-021-89854-5.

Win, T.T., Khan, S. and Fu, P., 2020. Fungus- (Alternaria sp.) mediated silver nanoparticles synthesis, characterization, and screening of antifungal activity against some phytopathogens. Journal of Nanotechnology, https://doi.org/10.1155/2020/8828878.

Wypij, M., Rai, M., Zemljič, L.F., Bračič, M., Hribernik, S. and Golińska, P., 2023. Pullulan-based films impregnated with silver nanoparticles from the Fusarium culmorum strain JTW1 for potential applications in the food industry and medicine. Frontiers in bioengineering and biotechnology, 11, p.1241739. https://doi.org/10.3389/fbioe.2023.1241739.

Xue, B., He, D., Gao, S., Wang, D., Yokoyama, K. and Wang, L., 2016. Biosynthesis of silver nanoparticles by the fungus Arthroderma fulvum and its antifungal activity against genera of Candida, Aspergillus and Fusarium. International journal of nanomedicine, 11, pp.1899–1906. https://doi.org/10.2147/IJN.S98339.

Yamaguchi, T., Tsuruda, Y., Furukawa, T., Negishi, L., Imura, Y., Sakuda, S., Yoshimura, E. and Suzuki, M., 2016. Synthesis of CdSe Quantum Dots Using Fusarium oxysporum. Materials (Basel, Switzerland), 9(10). https://doi.org/10.3390/ma9100855.

Yassin, M.A., El-Samawaty, A.E.-R.M.A., Dawoud, T.M., Abd-Elkader, O.H., Al Maary, K.S., Hatamleh, A.A. and Elgorban, A.M., 2017. Characterization and anti-Aspergillus flavus impact of nanoparticles synthesized by Penicillium citrinum. Saudi journal of biological sciences, 24(6), pp.1243–1248. https://doi.org/10.1016/j.sjbs.2016.10.004.

Zeng, Y.-J., Wu, X.-L., Yang, H.-R., Zong, M.-H. and Lou, W.-Y., 2023. 1,4-α-Glucosidase from Fusarium solani for Controllable Biosynthesis of Silver Nanoparticles and Their Multifunctional Applications. International Journal of Molecular Sciences, 24(6), p.5865. https://doi.org/10.3390/ijms24065865.

Zhu, Y., Hu, X., Qiao, M., Zhao, L. and Dong, C., 2024. Penicillium polonicum-mediated green synthesis of silver nanoparticles: Unveiling antimicrobial and seed germination advancements. Heliyon, 10(7), p.e28971. https://doi.org/10.1016/j.heliyon.2024.e28971.

Zielonka, A., Żymańczyk-Duda, E., Brzezińska-Rodak, M., Duda, M., Grzesiak, J. and Klimek-Ochab, M., 2018. Nanosilica synthesis mediated by Aspergillus parasiticus strain. Fungal biology, 122(5), pp.333–344. https://doi.org/10.1016/j.funbio.2018.02.004.

Zomorodian, K., Pourshahid, S., Sadatsharifi, A., Mehryar, P., Pakshir, K., Rahimi, M.J. and Arabi Monfared, A., 2016. Biosynthesis and Characterization of Silver Nanoparticles by Aspergillus Species. BioMed research international, 2016, p.5435397. https://doi.org/10.1155/2016/5435397.

**Table S3** – Fungal species employed in the biosynthesis of silver, gold and zinc nanoparticles: Precursor, morphology, and size.

| **Species** | **Precursor** | **Morphology** | **Size (nm)** | **References** |
| --- | --- | --- | --- | --- |
| *Alternaria sp.* | AgNO_3_ | Spherical | 3 to 10 | (Win, Khan and Fu, 2020) |
| *Arthroderma fulvum* |  |  | 15.5 ±2.5 | (Wang et al., 2016) |
| *Aspergillus clavatus* |  |  | 74.20 ± 21.20 | (Zomorodian et al., 2016) |
| *Aspergillus flavus* |  | Quasi-spherical | 88.1 | (Barapatre, Aadil and Jha, 2016) |
| *Aspergillus flavus* |  | Spherical | 117.0 ± 35.5 | (Zomorodian et al., 2016) |
| *Aspergillus fumigatus* |  |  | 49.0 ± 19.6 | (Zomorodian et al., 2016) |
| *Aspergillus fumigatus* |  |  | 17 to 75 | (Ottoni et al., 2019) |
| *Aspergillus fumigatus* |  |  | 3 to 13 | (Sarsar, Selwal and Selwal, 2016) |
| *Aspergillus Japonicus* |  |  | 3.8 to 23 | (Basheer et al., 2023) |
| *Aspergillus luchuensis* |  |  | 16 to 18 | (Abd Elghaffar et al., 2024) |
| *Aspergillus niger* |  |  | 3 to 13 | (Awad et al., 2022) |
| *Aspergillus niger* |  |  | 93.3 ± 28.3 | (Zomorodian et al., 2016) |
| *Aspergillus oryzae* |  |  | 3.8 to 23 | (Basheer et al., 2023) |
| *Aspergillus parasiticus* |  |  | 27.7 | (Ali, Rajendran and Abdallah, 2024) |
| *Aspergillus sp.* |  |  | 7.2 | (Abdelrahman et al., 2024) |
| *Aspergillus sydowii* |  |  | 1 to 24 | (Wang et al., 2021) |
| *Aspergillus terreus* |  |  | 10 to 18 | (Ammar and El-Desouky, 2016) |
| *Aspergillus terreus* |  |  | 21 to 25 | (Al-Enazi, Alsamhary and Ameen, 2024) |
| *Aspergillus terreus* |  |  | 7 to 23 | (Lotfy et al., 2021) |
| *Aspergillus terreus* |  |  | 3.8 to 23 | (Basheer et al., 2023) |
| *Aspergillus tubingensis* |  |  | 35 ± 10 | (Ottoni et al., 2019) |
| *Chaetomium thermophilum* |  |  | 9 | (Alves et al., 2022) |
| *Cladosporium halotorans* |  |  | 20 | (Ameen et al., 2023) |
| *Colletotrichum incarnatum* |  |  | 5 to 25 | (Chandankere et al., 2020) |
| *Emericella nidulan* |  | Hexagonal | 66.6 | (Barapatre, Aadil and Jha, 2016) |
| *Fusarium chlamydosporum* |  | Spherical | 6 to 26 | (Khalil, Abd El-Ghany and Rodríguez-Couto, 2019) |
| *Fusarium culmorum* |  |  | 15 | (Wypij et al., 2023) |
| *Fusarium oxysporum* |  |  | 34 | (Almeida, de Oliveira and Hotza, 2017) |
| *Fusarium oxysporum* |  |  | 21.3 | (Ahmed, Hamzah and Maaroof, 2018) |
| *Fusarium oxysporum* |  |  | 30 to 36 | (Ilahi et al., 2022) |
| *Fusarium scirpi* |  | Quasi-spherical | 2 to 20 | (Rodríguez-Serrano et al., 2020) |
| *Fusarium solani* |  | Spherical | 50 | (Zeng et al., 2023) |
| *Fusarium solani* |  |  | 5 to 30 | (Abd El-Aziz et al., 2015) |
| *Penicillium chrysogenum* |  |  | 9 to 18 | (Khalil, Abd El-Ghany and Rodríguez-Couto, 2019) |
| *Penicillium citreonigrum* |  | Quasi-spherical | 6 to 26 | (Hamad, 2018) |
| *Penicillium citrinum* |  | Spherical | 3 to 13 | (Yassin et al., 2017) |
| *Penicillium decumbens* |  |  | 70 | (Majeed et al., 2016) |
| *Penicillium expansum* |  | - | 14 to 25 | (Ammar and El-Desouky, 2016) |
| *Penicillium fimorum* |  | Spherical | 35 to 85 | (Barabadi et al., 2023) |
| *Penicillium italicum* |  | - | 33 to 46 | (Nayak, Nanda and Prabhakar, 2018) |
| *Penicillium oxalicum* |  | Spherical | 20 to 80 | (Bhattacharjee et al., 2017) |
| *Penicillium polonicum* |  |  | 3 to 25 | (Zhu et al., 2024) |
| *Penicillium simplicissimum* |  |  | 3.8 to 23 | (Basheer et al., 2023) |
| *Scopulariopsis brumptii* |  | Quasi-spherical | 4.2 to 23.2 | (Hamad, 2018) |
| *Alternaria sp.* | HAuCl_4_ | Spherical or quasi-spherical | 9.5±2.9 | (Olvera-Aripez et al., 2024) |
| *Aspergillus terreus* |  | Spherical | 9 to 14 | (Mishra et al., 2022) |
| *Aspergillus sydowii* |  |  | 9 to 16 | (Vala, 2015) |
| *Botrytis cinerea* |  | Spherical or quasi-spherical | 92.9±23.1 | (Olvera-Aripez et al., 2024) |
| *Cladosporium cladosporioides* |  |  | 60 | (M et al., 2017) |
| *Cladosporium oxysporum* |  | Quase-spherical | 72 | (Bhargava et al., 2016) |
| *Fusarium solani* |  | needle and flower-like structures with a spindle | 40 to 25 | (Clarance et al., 2020) |
| *Ganoderma sessile* |  | Spherical or quasi-spherical | 13.0±6 5.4 | (Olvera-Aripez et al., 2024) |
| *Penicillium citrinum* |  | Spherical | 60 to 80 | (Manjunath, Joshi and Raju, 2017) |
| *Penicillium rubens* |  |  | 15 | (Bhandari et al., 2023) |
| *Trichoderma asperellum* |  | Spherical or quasi-spherical | 16.4±4.0 | (Olvera-Aripez et al., 2024) |
| *Trichoderma atroviride* |  |  | 24.7±7.6 | (Olvera-Aripez et al., 2024) |
| *Tricholoma crassum* |  | circular, rhomboid (<5 nm), hexagonal, cuboidal, triangular | 2–22 nm (most 5–10 nm) | (Basu et al., 2018) |
| *Aspergillus fumigatus* | ZnNO₃·6H₂O | flowerlike zinc phosphate crystal structure with an irregular nanosheet morphology | - | (El-Sharkawy and Abbas, 2023) |
|  |  |  |  |  |
| *Aspergillus niger* | Zn(CH_3_COO)_2_·_2_H_2_O | Distorted spherical | 10 to 70 | (Es-Haghi et al., 2021) |
|  |  |  |  |  |
| *Aspergillus niger* | Zn(CH₃COO)_2_ | Spherical | 31.75 ± 4.38 | (Abdelkader et al., 2022) |
|  |  |  |  |  |
| *Aspergillus terreus* |  |  | 10 to 70 | (Chen et al., 2021) |
|  |  |  |  |  |
| *Cordyceps militaris* | Zn(CH_3_COO)_2_·2H_2_O |  | 7 to 16 | (Dias et al., 2022) |
|  |  |  |  |  |
| *Penicillium griseofulvum* |  |  | 46 to 82.1 | (Li, Wu and Zhang, 2025) |
|  |  |  |  |  |
| *Phanerochaete chrysosporium* | ZnSO_4_·_7_H_2_O | Hexagonal | 5 to 200 | (Sharma, Dhayal and Sharma, 2021) |
